# Supplementary material for: Intrinsic group behaviour II: On the dependence of triad spatial dynamics on social and personal features; and on the effect of social interaction on small group dynamics
Source: PLoS One. 2019 Dec 3;14(12):e0225704. doi: 10.1371/journal.pone.0225704 (PMC6890182; doi:10.1371/journal.pone.0225704)
Supplement: S1 Annex — (ZIP) [file pone.0225704.s005.zip › annex_1/interaction_coding.pdf]

歩行者ID (pedestrian ID)	Gサイズ (group size)	同行ID (partner ID)	相手サイズ (number of interaction partners)	相手ID (interaction partner ID)	備考 (notes)	視線：相手を見た (gaze: watched the partner)	会話中にしか出ないような身振り、口の動きがあった (behaviours seen only during conversation, such as moving the mouth)	視線：同じ方向を見た (gaze: watched in the same direction)	フィジカルコンタクト (physical contact)	その他 (other)	判断つかず（根拠はないけど会話しているように見える）(not used: looks like they are having a conversation but it is impossible to judge)	メモ (notes)
10050100	2	10045500	1	10045500		○	○					
10045500	2	10050100	1	10050100		○						
10061100	2	10063001	1	10063001		○						
10063001	2	10061100	1	10061100								
10093200	2	10100800	1	10100800		○						
10100800	2	10093200	1	10093200								
10140500	2	10140400	1	10140400		○				○		
10140400	2	10140500	1	10140500		○				○		
10191700	2	10191501	1	10191501		○						
10191501	2	10191700	1	10191700		○						
10182000	2	no ID	1	no ID		○				○		
no ID	2	10182000	1	10182000		○				○		
10202000	2	10202100	1	10202100			○					
10202100	2	10202000	1	10202000		○						
10211200	2	10211600	0	0								
10211600	2	10211200	0	0								
10221800	3	10204500,	2	10204500, 10222000		○	○					
10204500	3	10221800,	2	10221800, 10222000		○	○					
10222000	3	10221800,	2	10221800, 10204500		○	○					
10244900	2	10245101	0	0								
10245101	2	10244900	0	0								
10251900	3	10251700,	0	0								
10251700	3	10251900,	0	0								
10251902	3	10251900,	0	0								
10251400	2	10252800	1	10252800		○	○			○		
10252800	2	10251400	1	10251400		○				○		
10214900	2	10233500	1	10233500		○	○					
10233500	2	10214900	1	10214900								
10280001	2	10275200	0	0								
10275200	2	10280001	0	0								
10294900	2	10294700	1	10294700		○						
10294700	2	10294900	1	10294900								
10322100	2	10322701	0	0								
10322701	2	10322100	0	0								
10341600	2	10341700	1	10341700		○						
10341700	2	10341600	1	10341600		○						
10363000	2	10362900	1	10362900				○				
10362900	2	10363000	1	10363000			○	○				
10382601	2	10383800	1	10383800			○	○				
10383800	2	10382601	1	10382601				○				
10403500	2	10403800	1	10403800					○			
10403800	2	10403500	1	10403500								
10403100	2	10405300	0	0								
10405300	2	10403100	0	0								
10414300	2	10414000	1	10414000					○			
10414000	2	10414300	1	10414300					○			
10420101	4	10420103,	0	0								
10420103	4	10420101,	0	0								
10420300	4	10420101,	1	10420400		○						
10420400	4	10420101,	1	10420300								
10445801	3	10442300,	2	10442300, 10442800		○						
10442300	3	10445801,	2	10445801, 10442800		○			○			

10442800	3	10445801,	2	10445801, 10442300	○					
10444900	2	10444800	0	0						
10444800	2	10444900	0	0						
10465201	2	10465103	1	10465103	○					
10465103	2	10465201	1	10465201						
10465102	2	10465200	1	10465200	○					
10465200	2	10465102	1	10465102	○					
10473400	3	10473300,	1	10473300	○		○		○	
10473300	3	10473400,	2	10473400, 10464900	○		○		○	
10464900	3	10473400,	1	10473300			○			
10464901	3	10471400,	2	10471400, 10464400		○	○			
10471400	3	10464901,	2	10464901, 10464400			○			
10464400	3	10464901,	2	10464901, 10471400			○			
10472100	2	10472200	0	0						
10472200	2	10472100	0	0						
10481201	2	10481600	1	10481600	○					
10481600	2	10481201	1	10481201						
10491400	2	10491500	1	10491500	○	○				
10491500	2	10491400	1	10491400						
10534301	5	10534300,	1	10534300	○	○				
10534300	5	10534301,	1	10534301						
10534500	5	10534301,	0	0						
10534401	5	10534301,	0	0						
10534400	5	10534301,	0	0						
10552100	2	10552600	1	10552600	○					
10552600	2	10552100	1	10552100	○					
10553100	2	10552900	1	10552900	○					
10552900	2	10553100	1	10553100						
10560202	2	10560400	1	10560400						
10560400	2	10560202	1	10560202	○	○				
10564000	2	10563700	0	0						
10563700	2	10564000	0	0						
10551100	2	10560201	1	10560201	○	○				
10560201	2	10551100	1	10551100	○					
10570702	2	10570701	1	10570701				○		
10570701	2	10570702	1	10570702				○		
10575500	2	10572500	1	10572500	○			○	○	
10572500	2	10575500	1	10575500	○			○	○	
10580300	2	10575400	1	10575400					○	
10575400	2	10580300	1	10580300					○	先に店に入
12005700	2	12005600	1	12005600	○					
12005600	2	12005700	1	12005700	○					
12015500	2	12015300	1	12015300						
12015300	2	12015500	1	12015500		○				
12024200	2	12023800	1	12023800	○					
12023800	2	12024200	1	12024200	○					
11583000	2	12024900	1	12024900	○					
12024900	2	11583000	1	11583000	○					
12025200	2	12025100	1	12025100	○	○				
12025100	2	12025200	1	12025200	○					
12040200	2	12040500	1	12040500		○				
12040500	2	12040200	1	12040200		○	○			
12041400	2	12041200	1	12041200	○					
12041200	2	12041400	1	12041400	○					
12042500	2	12042200	1	12042200						
12042200	2	12042500	1	12042500	○	○				
12024600	2	11583200	1	11583200	○	○				
11583200	2	12024600	1	12024600	○					
12050000	2	12045500	1	12045500	○	○				
12045500	2	12050000	1	12050000						
