# Supplementary material for: Intrinsic group behaviour II: On the dependence of triad spatial dynamics on social and personal features; and on the effect of social interaction on small group dynamics
Source: PLoS One. 2019 Dec 3;14(12):e0225704. doi: 10.1371/journal.pone.0225704 (PMC6890182; doi:10.1371/journal.pone.0225704)
Supplement: S1 Annex — (ZIP) [file pone.0225704.s005.zip › annex_1/interaction_criteria.pdf]

【対象】(Coded as interacting)

・一人が手を、通常の垂らして前後に動かす歩くときの動作ではなく、胸の高さまで掲げる動作があった。(one of them moved one hand up to the chest, in a way that goes beyond the usual movement during walking)

・同じ方向（進行方向とは違う）を見ながら、一人が見ている方を指さした (they watched in the same direction, different from the walking one, and one of them pointed in such direction)

・母子で手をつないでいて、つないでいる手をひょいっと上げた。(A parent and child were holding hands, and suddendly raised their hands)

・一人が腕を通常より高く上げて指差しなどジェスチャをし、そのタイミングで二人同じ方向を見た。(one of them raised an arm higher than usual making a pointing gesture, and at that point they watched in the same direction at the same time)

・一人が先に立ち止まり、先に行きかけたもう一人も戻ってきて、指差すようなジェスチャをしながらしばらく止まっていた。目線が合うことはなかった。(One person stopped, and the other person, who was walking, stopped and came back, while making pointing gestures; even if there was no gaze contact)

・二人して、同じ方向を指差しながら、同じ方向を見た。(they watched in the same direction while making pointing gestures)

・一人が、片腕をあげて指差し（腕だけの場合も）をした。(one of them made a pointing gesture with an arm, even if just using the arm)

・曲がり角などでない、まっすぐな道で、二人同じタイミングで同じ方向（横）を見て、同じタイミングで前を向いた。目線が合うことはなかった。(while walking straight in an area without turns, they watched at the same time in the same direction on the side, and then watched again towards the walking direction at the same time, even if there was no gaze contact)

・同じタイミングで同じ方向を見ることが、10 秒以内に 2 回以上あった。(in less than 10 seconds watched at least twice in the same direction at the same time)

・腕を振り上げるジェスチャがあり、また、顔の表情が大きく動き、何か喋っているように見えた。(there were arm gestures, face expression was changing, and looked like they were talking)

・二人同じタイミングで立ち止まり引き返した。(they stopped and went back at the same time)

・一人が立ち止まり、周りを見回して、指差しのようなジェスチャをした。もう一人はビデオに映っていないが、HumanPosition ではすぐ近くにいる。(one of them stopped, looked around, made pointing gestures; the other person stayed close; although the second person was not visible in the video and the position information was given only by the visualisation of tracked trajectories)

・一人が立ち止まり、しばらく周りを見回した後、再び歩き出した。もう一人はビデオに映っていないが、HumanPosition では、同じタイミングで立ち止まり、歩き出した。。(one of them stopped, looked around, and then started walking again; the other person stopped and walked at the same time; although the second person was not visible in the video and the position information was given only by the visualisation of tracked trajectories)

・視線を若干相手寄りに向け、腕を通常より高くあげて何かジェスチャした。(one person gazed briefly in the direction of the partner, and made some gesture raising the arm)

・HumanPositionでは、途中まで一緒に歩いてきて、十字路で別れた。別れ際、一人が（相手の方へ顔を向けて？ビデオには映っていない）手を振った。(They walked together for a while, as shown by the visualisation of tracked trajectories, and then at a crossing they split; at the moment of splitting they waved hands; gaze and face direction information was not available)

・一人が指差しをし、一緒に方向転換した。(one of them pointed to something, and they changed direction at the same time)

・曲がり角などで、二人同じタイミングで同じ方向（横）を見て、同じタイミングで道を曲がった。口や表情も動いているように見えた。目線が合うことはなかった。(at a turning point they watched towards the same direction on the side, and turned at the same time. There was a movement of the mouth and changing expression, even if no gaze contact)

・案内板を一緒に見ていた。(they watched together the information board)

・お互いに同時に相手の荷物を見た。一人が右手を胸下あたりまで上げて、相手に合図？した。(they watched at the same time one's baggage, one of them raised slightly the right hand, and probably made a gesture towards the other)

・車いすと付添人で、車いすの人が腕をしきりに動かしてジェスチャしていた。(A person on a wheelchair moved many times one arm, making a gesture towards another person)

・二人一緒に、立ち止まり、同じ場所でしばらく逡巡した後、歩き出した。(two people stopped at the same time, hesitated for a while around the same place, and then started walking again)

・二人ほぼ同じタイミングで首を傾げる動作をした。(Leaned their heads almost at the same time)

・一人が、もう一人の肩に手をおいた。(one of them put a hand on the other's shoulder)

・目線はあわせていないが、顔を少し寄せているように見えたのと、一人が拳を顔の前にかざすような動作をした。(Although there was no gaze contact, it looked like they drew their face closer, one of them raised one hand at the height of the face)

【対象外】(Not coded as interacting)

・それぞれに、きょろきょろと周りを見回している。同じ方向を見たり、視線を交わすことは無い。(They watched around curiously, in an independent way; they never looked at each other or in the same direction)

・ただ歩いているだけでは出ないくらい手腕が胸あたりまで高くあがったが、相手全身と本人が半分ほど見切れていて判断に迷ったため除外。(Their hands were at chest level, higher than during normal walking, but they were partially or completely obstructed and it was impossible to judge)

・相手との背丈に差があり（相手が低い）、目線は相手の方向を向いていても、その向こうを見ているような場合。(one of them was much taller, watched in the direction of the other, but appeared to watch over the other's head)

・同じタイミングで同じ方向を見たが、歩く位置が前後にずれている、ちょうど十字路に出たところで反射的に見たのかも、同じ方を見る時間が短く人同士が少し離れて歩いていた、など、会話しているように見えなかった場合。(they watched in the same direction at the same time, but their walking was not aligned, they watched in the same direction after a crossing, they watched in the

same direction for short time, they were not close and similar; they did not look as having an interaction)

・小首を傾げるなどの動作が動作が小さく、確信が持てない場合。(They leaned their heads but very little, no judging confidence)

・車いすと付添人の場合、付添人が体を曲げた状態をキープするなど明らかに会話している様子を見て取れる場合以外は、除外した。(When a person was on a wheelchair, only clear conversation hints such as the walking companions clearly bending their body for a relatively long time were considered)

・かばんの持ち手を肩にかけ直す、髪を触る、扇子を出してあおいだり、両手を胸の前で組むなど、コミュニケーションに関係ないと思われるジェスチャは、除外した。(All gestures unrelated to communication, such as moving an arm to correct the position of a bag, touching the hair, using a fan, folding arms or similar were not considered)
