# Supplementary material for: Intrinsic group behaviour II: On the dependence of triad spatial dynamics on social and personal features; and on the effect of social interaction on small group dynamics
Source: PLoS One. 2019 Dec 3;14(12):e0225704. doi: 10.1371/journal.pone.0225704 (PMC6890182; doi:10.1371/journal.pone.0225704)
Supplement: S1 Annex — (ZIP) [file pone.0225704.s005.zip › annex_1/readme.pdf]

Four coders (coder A, coder B, coder C and coder D) were involved in the coding process. All of them were non-technical staff members of ATR. They were aware, as part of their working contracts, that their coding would be used for scientific research and publications. Consent regarding these specific coding tasks was reached verbally.

Coders A and B performed the group and interaction coding for the cited work [3]. Instructions to them were given verbally by the corresponding author, FZ, in Japanese. The indications were kept by intention as little specific as possible, such as "use visual clues and common sense to understand if pedestrians belong to a group, and if they are explicitly socially interacting during the observed time".

As the coders were having some difficulties in coding the presence of social interaction, author TK suggested them to check if at least one of the following was happening

- gaze: watched the partner
- behaviours seen only during conversation, such as moving the mouth
- gaze: watched in the same direction
- physical contact
- other (possible clear sign of social interaction, based on common sense)

Coder A performed also the interaction coding used for the cited work [4] and for the current work. She wrote down in detail her personal criteria for coding interaction, which are shown along with an English translation in the attached document interaction *criteria.pdf*. An example of her coding files may be found in *interaction_coding.xlsx* (coders operated on excel files, these files have been analysed for internal consistency using a C++ code written by corresponding author FZ, and only consistent data was included in the data set).

Coder B instructed coder C (used for the main coding of cited work [46] and for cross-evaluation in the present manuscript) and coder D (used for the present work). The instructions of coder B to coder D may be found, along with an English translation, in *3_people_instructions.pdf*, while an example of coder Ds coding is in *3_people_coding.xlsx*.

All translations from Japanese to English have been done by corresponding author FZ.
