# Supplementary material for: Intrinsic group behaviour II: On the dependence of triad spatial dynamics on social and personal features; and on the effect of social interaction on small group dynamics
Source: PLoS One. 2019 Dec 3;14(12):e0225704. doi: 10.1371/journal.pone.0225704 (PMC6890182; doi:10.1371/journal.pone.0225704)
Supplement: S1 Annex — (ZIP) [file pone.0225704.s005.zip › annex_1/3_people_instructions.pdf]

商業施設内を通行するグループに対し、下記の項目を分類していただきます。
Divide pedestrian groups moving in a public space according to the categories below
・3 人のグループで行動する通行人がコーディングの対象です。
The target of coding is groups composed of 3 people (*translator note: the size of groups had already been coded by a previous coder*)
・判断基準は一定でお願いします。
Please use consistent coding criteria

＜相手サイズ＞ Number of (*interaction*) partners
同グループ内でインタラクションした人の人数を入力
It shows the number of people each pedestrian was socially interacting with (*translator note: the number of interaction partners had already been coded by a previous coder*)

＜グループの関係＞ Group relation
0　判別不可能 Not able to judge
1　恋人 couple
2　同僚 colleagues
3　家族 family
4　友人 friends

＜年齢＞ age
年代を入力して下さい。Insert the age in decades
10 才未満の小さなお子さんについては、思う年齢を入力して下さい。For children below 10 years old, try to guess the age in years

＜性別＞ Gender
0　女 Female
1　男 Male

＜用事＞ Purpose
0　遊び Leisure
1　仕事 Work

＜詳しい説明＞ Detailed explanation
・対象者がベビーカー、車イスを使用している場合は必ず記載して下さい。
Specify if the pedestrians are using strollers or wheelchairs

・対象者の関係が分かりにくい場合や他の人に被って判断できない場合など

In case the relation is hard to understand, or if the pedestrian is obstructed and not clearly visible

　判断しにくい場合については、その旨を記載し、当該IDの服装なども

　わかるよう記載して下さい

In such a case write it down explicitly, along with the pedestrian ID and a description of the pedestrian appearance (clothes etc.)
