# Supplementary material for: Intrinsic group behaviour II: On the dependence of triad spatial dynamics on social and personal features; and on the effect of social interaction on small group dynamics
Source: PLoS One. 2019 Dec 3;14(12):e0225704. doi: 10.1371/journal.pone.0225704 (PMC6890182; doi:10.1371/journal.pone.0225704)
Supplement: S1 Appendix — (PDF) [file pone.0225704.s006.pdf]

Coder reliability

Analysis of coder agreement

We consider two possible statistical indicators of agreement between coders.

Cohen's κ

Cohen's κ [1] is a very popular indicator to compare the agreement between two coders, based on the equation

$$\kappa = \frac{p - p_r}{1 - p_r}, \tag{1}$$

where p stands for the agreement rate between coders and p_r for the probability of random agreement. The agreement between coders is shown in Table 1.

Table 1: Agreement between coders according to Cohen's κ statistics.

Purpose	Relation	Gender	Min Age
0.873	0.894	0.958	0.476

These results show that in general the agreement is higher for gender, followed by relation and purpose[1]. Although there is no real sound mathematical way to evaluate the absolute value of these numbers, according to popular benchmarks, an agreement between 0.8 and 1 is considered as "almost perfect", an agreement between 0.6 and 0.8 as "substantial", while an agreement between 0.4 and 0.6 is only "moderate" [2]. The agreement on age is not very good. It has to be noticed that in Cohen's κ statistics, the difference between the values assigned by the coders is not taken in account (all coded values are considered "nominal" and there is no way to evaluate the difference between them). To take this into account, we can use Krippendorff's α statistics.

Krippendorff's α

The Krippendorff's α statistics [3], that allows for consideration of quantitative differences between coding results, gives the results shown in Table 2.

Table 2: Agreement between coders according to Krippendorff's α statistics. Purpose, and relation are "nominal" data, gender is ordinal ("number of females"), and age is on an "interval", according to the definition of α statistics.

Purpose	Relation	Gender	Min Age
0.873	0.895	0.966	0.818

[1] For relation, we excluded the data on which there was no coding by one of the coders, which reduced the sample and could be the reason for relation over-performing the apparently "easier" task of coding purpose.

Krippendorff does not provide any "magic number" but suggests to require $\alpha > 0.8$, satisfied by all categories, for reliable results.

Discussion

Using popular indicators of coder reliability, we have found that, in relative terms, the most reliable coding regards gender, followed by relation and purpose. In absolute terms, according to the Krippendorff's α statistics that can better cope with the nature of our data, we may see that all codings may be considered as sufficiently reliable to provide sound findings.

Quantitative comparison of results

The analysis based on the above indicators provides an estimate on the reliability of coding of pedestrians in different categories. We may nevertheless use another approach to test the reliability of our findings when based on different coding processes. Since for each category we analyse the values of the observables V, r, x and y, we may compare these quantitative results between different coders.

We perform this comparison, which has also the advantage of being based on more mathematically sound statistical indicators (standard errors, ANOVA analysis) for relation and gender, since these two categories are the main focus of this work.

Relation

The results (on the common subset of data) for the relation dependence of all observables between the main coder (coder 1) and the secondary coder (coder 2) are compared in Tables 3 and 4. Despite the reduced samples, the only observable that presents a difference between the two coders which is larger than the standard error is x in the family category (the difference between the two averages being 75 mm, with standard errors of 41 and 39 mm). Nevertheless, such difference does not affect the result according to which families present a lower value of x in a statistically significant way (it may be noticed that coder 2 was not able to identify the relation properties of 14 triads).

Table 3: Observable dependence on relation for triads according to coder 1 (common data set only). Lengths in millimetres, times in seconds.

Relation	N_g^k	V	r	x	y
Colleagues	70	1200 ± 18 (σ=155)	600 ± 15 (σ=129)	1120 ± 31 (σ=258)	593 ± 42 (σ=349)
Families	38	1069 ± 28 (σ=176)	594 ± 23 (σ=144)	939 ± 41 (σ=253)	612 ± 57 (σ=354)
Friends	55	1040 ± 21 (σ=155)	581 ± 19 (σ=137)	1076 ± 37 (σ=275)	550 ± 43 (σ=317)
$F_{2,160}$		17.2	0.299	5.85	0.426
p		$1.66 \cdot 10^{-7}$	0.742	0.00355	0.654
R^2		0.177	0.00373	0.0681	0.00529
δ		1.03	0.143	0.707	0.188

Table 4: Observable dependence on relation for triads according to coder 2 (common data set only). Lengths in millimetres, times in seconds.

Relation	N_g^k	V	r	x	y
Colleagues	63	1212 ± 19 (σ=154)	599 ± 17 (σ=133)	1127 ± 30 (σ=238)	579 ± 45 (σ=356)
Families	30	1079 ± 34 (σ=185)	601 ± 28 (σ=154)	864 ± 39 (σ=212)	652 ± 64 (σ=351)
Friends	59	1046 ± 20 (σ=153)	582 ± 18 (σ=136)	1086 ± 39 (σ=296)	549 ± 41 (σ=312)
$F_{2,149}$		17.3	0.272	10.8	0.911
p		$1.72 \cdot 10^{-7}$	0.762	$4.03 \cdot 10^{-5}$	0.405
R^2		0.189	0.00364	0.127	0.0121
δ		1.08	0.134	1.14	0.317

Gender

The results (on the common subset of data) for the gender dependence of all observables between the main coder (coder 1) and the secondary coder (coder 2) are compared in Tables 5 and 6. Despite the relatively small samples, differences between average values due to the different codings are always small when compared to standard errors, showing the reliability of gender coding.

Table 5: Observable dependence on gender for triads according to coder 1 (common data set only). Lengths in millimetres, times in seconds.

Gender	N_g^k	V	r	x	y
Three females	42	1061 ± 22 (σ=142)	565 ± 18 (σ=119)	1036 ± 38 (σ=249)	548 ± 48 (σ=313)
Two females	27	1018 ± 28 (σ=146)	589 ± 34 (σ=176)	1061 ± 60 (σ=310)	524 ± 78 (σ=407)
Two males	24	1044 ± 31 (σ=150)	610 ± 22 (σ=108)	1005 ± 42 (σ=207)	675 ± 46 (σ=224)
Three males	70	1211 ± 20 (σ=167)	603 ± 16 (σ=134)	1100 ± 34 (σ=285)	595 ± 42 (σ=354)
$F_{3,159}$		15.4	0.854	0.923	1.03
p		$< 10^{-8}$	0.467	0.431	0.38
R^2		0.226	0.0159	0.0171	0.0191
δ		1.19	0.39	0.356	0.455

Table 6: Observable dependence on gender for triads according to coder 2 (common data set only). Lengths in millimetres, times in seconds.

Gender	N_g^k	V	r	x	y
Three females	43	1059 ± 21 (σ=141)	572 ± 19 (σ=123)	1036 ± 39 (σ=255)	551 ± 50 (σ=329)
Two females	24	1013 ± 30 (σ=145)	594 ± 35 (σ=174)	1100 ± 61 (σ=297)	511 ± 81 (σ=398)
Two males	24	1038 ± 31 (σ=154)	594 ± 25 (σ=120)	984 ± 43 (σ=210)	655 ± 46 (σ=227)
Three males	72	1210 ± 19 (σ=165)	603 ± 16 (σ=132)	1093 ± 34 (σ=285)	602 ± 41 (σ=352)
$F_{3,159}$		16.1	0.462	1.25	0.913
p		$< 10^{-8}$	0.709	0.294	0.436
R^2		0.233	0.00864	0.023	0.0169
δ		1.23	0.24	0.449	0.446

References

[1] Cohen, Jacob *A coefficient of agreement for nominal scales*. Educational and Psychological Measurement 20 (1): 3746 (1960).

[2] Landis, J.R.; Koch, G.G. (1977). *The measurement of observer agreement for categorical data* Biometrics. 33 (1): 159174.

[3] Krippendorff, Klaus. *Reliability in content analysis*, Human communication research 30.3 (2004): 411-433.
