# Supplementary material for: Intrinsic group behaviour II: On the dependence of triad spatial dynamics on social and personal features; and on the effect of social interaction on small group dynamics
Source: PLoS One. 2019 Dec 3;14(12):e0225704. doi: 10.1371/journal.pone.0225704 (PMC6890182; doi:10.1371/journal.pone.0225704)
Supplement: S2 Appendix — (PDF) [file pone.0225704.s007.pdf]

Statistical analysis of observables

In this work we are interested in describing how pedestrian group behaviour is influenced by some *intrinsic features*, such as purpose, relation, gender, age or height. Each feature (or factor) may be divided into k categories (e.g., in the case of gender composition of triads $k = 4$ and the categories are no females, one female, two females or three females). Each group is coded as belonging to a specific category, so that each category has N_g^k groups. As described in section "Materials and methods-Data set-Trajectories", for each group $i \in N_g^k$ we can measure the value of observable o every 500 ms. We may call these measurements $o_{i,j}^k$ with $j = 1, \ldots, n_i^k$ (i.e. we have n_i^k measurements, or events, corresponding to group i in category k).

We believe that the largest amount of quantitative information regarding the dependence of group behaviour on intrinsic features is included in the overall probability distributions functions concerning all $N^k = \sum_{i \in N_g^k} n_i^k$ measurements of a given observable, as shown for example in Figure 3 in the main text, since from the analysis of these figures, we can understand what is the probability of having a given value for each observable in each category.

It is nevertheless useful to extract some quantitative information, such as average values and standard deviations, from these distributions. Furthermore, although the purpose of this paper is not to provide a "p-value statistical independence label" to each feature; to compare such average values it is customary and useful to compute, along with other statistical indicators such as effect size and determination coefficient, the standard error of each distribution and to perform the related analysis of variance (ANOVA). The computation of these latter statistical quantities is nevertheless based on an assumption of statistical independence of the data, an assumption that clearly does not hold for all our N^k observations[1].

[1] As an extreme case, we can imagine that for a given k we were following a single group ($N_g^k = 1$) for one hour ($n_1^k = 7200$). We will have then, if we ignore measurement noise, a perfect information regarding the behaviour of that group in that hour and, under the strong assumption of time independence in the group behaviour, a good statistics about the behaviour *of that particular group*. We still do not have any information about how group behaviour changes between groups in the category, since that information depends on the number of groups analysed, N_g^k. Furthermore, since in general we track a given group only for the few seconds it needs to cross the corridor, the observations $o_{i,j}$ at fixed i are also strongly time correlated.

Average values, standard deviations and standard errors

We thus proceed in the following way, justified by having a similar number of observations for each group[2]. For each observable o we compute the average over group i

$$O_i^k = \frac{\sum_{j=1}^{n_i^k} o_{i,j}^k}{n_i^k}, \tag{1}$$

and then provide its average value in the category k as

$$< O >_k \pm\varepsilon_k, \tag{2}$$

where $< O >$ and the standard error ε are given by

$$< O >_k = \frac{\sum_{i=1}^{N_g^k} O_i^k}{N_g^k}, \tag{3}$$

$$\varepsilon_k = \frac{\sigma_k}{\sqrt{N_g^k}}, \tag{4}$$

and the standard deviation is

$$\sigma_k = \sqrt{\frac{\sum_{i=1}^{N_g^k} (O_i^k)^2}{N_g^k} - < O >_k^2}. \tag{5}$$

As a rule of thumb, we may say that o assumes a different value between categories k and j provided that

$$|< O >_k - < O >_j | \gg 2\max(\varepsilon_k, \varepsilon_j). \tag{6}$$

Analysis of variance

This rule of thumb is obviously related to the ANOVA analysis reported in the main text. The ANOVA analysis proceeds as follows. We define n^c as the number of categories for a given feature,

$$N = \sum_{k=1}^{n^c} N_g^k, \tag{7}$$

as the total number of groups, and the overall average of the observable as

$$< O > = \frac{\sum_{k=1}^{n^c} < O >_k N_g^k}{N}. \tag{8}$$

[2]An average of 49 observations with a standard deviation of 22 over 1168 groups in the dyadic set, and an average of 32 observations with a standard deviation of 25 over 918 groups in the triadic one. We nevertheless exclude from the following analysis groups that provided less than 10 observation points, which has a stronger effect on the triadic set, eliminating a few groups with a small number of observations.

We then define the distance between $< O >$ and $< O >_k$ as

$$d_k =< O > - < O >_k, \tag{9}$$

and the degrees of freedom

$$\gamma_1 = n^c - 1, \qquad \gamma_2 = N - n^c. \tag{10}$$

The F factor is then defined as

$$F = \frac{\left(\sum_{k=1}^{n^c} d_k^2 N_g^k\right)}{\left(\sum_{k=1}^{n^c} \sigma_k^2 N_g^k\right)} \frac{\gamma_2}{\gamma_1}. \tag{11}$$

This result is reported in our tables as F_{γ_1,γ_2}, along with the celebrated p-value, that provides the probability, under the hypothesis of independence of data, that the difference between the distributions is due to chance [1]

$$p = 1 - \int_0^F f_{\gamma_1,\gamma_1}(x)dx. \tag{12}$$

The f distribution has to be computed numerically [2], but a value $F \gg 1$ assures a small p-value.

Let us see how this relates to the rule of thumb for standard errors. Let us assume we have two categories with the same number of groups for category

$$N_g^1 = N_g^2 = N_g. \tag{13}$$

We clearly have

$$< O >= \frac{< O >_1 + < O >_2}{2}, \tag{14}$$

$$|d_1| = |d_2| = \frac{|< O >_1 - < O >_2|}{2}, \tag{15}$$

and

$$F = \frac{|< O >_1 - < O >_2|^2}{\sigma_1^2 + \sigma_2^2}(N_g - 1). \tag{16}$$

Using[3]

$$\frac{\sigma_i^2}{N_g - 1} \approx \varepsilon_i^2, \tag{17}$$

we get the expression

$$F \approx \frac{|< O >_1 - < O >_2|^2}{\varepsilon_1^2 + \varepsilon_2^2} > \frac{|< O >_1 - < O >_2|^2}{(2\max(\varepsilon_1, \varepsilon_2))^2}, \tag{18}$$

so that the rule of thumb eq. 6 corresponds to have a high F-value and thus a low p-value.

[3] The actual definition of the standard error uses $\sqrt{N_g - 1}$ but the numbers shown in the tables use the approximate definition $\sqrt{N_g}$. For $N_g \approx 100$ or more, as it is usually the case in this work, the difference is at most 5%.

Coefficient of determination

Eq. 11 says that the F factor is high if the σ_k are smaller than the d_k, i.e. if the variation inside the categories are smaller than outside the category, and if the total number of observation is high. Due to the large number of data points, the F-values in appendix S2 (where we use all the observable measurement instead of group averages) are always very high, and the corresponding p-values very low, but the hypothesis of statistical independence of data underlying the usual interpretation of p is obviously not valid. There are nevertheless some statistical estimators that do not depend dramatically on the number of observations, and that will thus have a similar value either if performed using all the data points or if performed using only group averages.

One such estimator is the coefficient of determination

$$R^2 = 1 - \frac{\sum_{i,k}(o_i^k - < O >_k)^2}{\sum_{i,k}(o_i^k - < O >)^2}, \tag{19}$$

which can also be computed as from the F factor as

$$R^2 = \frac{F\gamma_1}{F\gamma_1 + \gamma_2}, \tag{20}$$

and provides an estimate of how much of the variance in the data is "explained" by the category averages.

Effect size

The R^2 coefficient may attain low values if two or more category distribution functions are very similar, as it is usually the case in our work. To point out the presence of at least one distribution that is clearly different from the others we may use the following definition of the effect size δ (δ-value). We first define [3]

$$\delta_{k,l} = \frac{< O >_k - < O >_l}{\overline{\sigma}}, \qquad \overline{\sigma} = \sqrt{\frac{(\tilde{n}_k - 1)\sigma_k^2 + (\tilde{n}_l - 1)\sigma_l^2}{\tilde{n}_k + \tilde{n}_l - 2}}, \tag{21}$$

where $\tilde{n}_k$, $\tilde{n}_l$ are the number of points used for computing the averages and standard deviations[4], and then we consider the maximum pairwise effect size

$$\delta = \max_{k,l} |\delta_{k,l}|. \tag{22}$$

While a p-value tells us about the significance of the statistical difference between two distributions, the difference may be often so small that it can be verified only if a large amount of data are collected. But if we have also $\delta \approx 1$, then the two distributions are different enough to be distinguished also using a relatively reduced amount of data.

[4] I.e., $\tilde{n}_k = N_g^k$ if we are using group averages, $\tilde{n}_k = N^k$ if we are using overall distributions.

Multi-factor cross analysis

We refrain from applying the machinery of two way or n way ANOVA to our data, since our ecological data set is unbalanced, and it is unbalanced for the very reason that our "factors" are not independent variables[5].

It is nevertheless useful to analyse the interplay between the different features, and we do that in appendix S3 by performing a statistical analysis similar to the one described above of a given feature A while keeping fixed the value of another feature B to a category $\overline{k}$.[6] Sometimes this analysis is performed on a reduced number of groups, and thus the corresponding p-value may be high. This does not imply that the analysis is valueless, at least in our opinion, since it provides new information. The F and p-values are, in this situation, useful to compare different observables on the given condition. Furthermore, in these situations, an analysis of statistical indicators that do not depend critically on the number of observations, such as the effect size, is particularly valuable.

References

[1] R. Ash, *Statistical Inference, a Concise Course*, Dover 2011

[2] W. Press, S. Teukolsky, W. Vetterling, B. Flannery, *Numerical Recipes in C*, Second edition, Cambridge University Press, 1992

[3] J. Cohen, *Statistical power analysis for the behavioural sciences*, Second edition, Routledge, 1988

[4] http://www.mext.go.jp/b_menu/toukei/001/022/2004/002.pdf (in Japanese)

[5] Zanlungo, F., Yücel, Z., Brščić, D., Kanda, T, and Hagita, N., *Intrinsic group behaviour: Dependence of pedestrian dyad dynamics on principal social and personal features*, PLoS one, 12, 11, e0187253 (Public Library of Science, 2017)

[5] For example, since the average height of females is two standard deviations lower than the male one [4], the high range height groups will be entirely composed of males, not to mention more extreme cases, such as the conditional probability of having a children in a group of colleagues, which is arguably zero.

[6] For the fixed category feature B, we use also the external feature of pedestrian crowd density. Since the same group may contribute to different densities, when operating at a fixed density we use for group averages all groups that contribute with at least 10 data points to the observable distribution for that density value (in [5] we required only 5 points, but in this work we used the same threshold, 10, used for observable computations at arbitrary densities).
