# Supplementary material for: Intrinsic group behaviour II: On the dependence of triad spatial dynamics on social and personal features; and on the effect of social interaction on small group dynamics
Source: PLoS One. 2019 Dec 3;14(12):e0225704. doi: 10.1371/journal.pone.0225704 (PMC6890182; doi:10.1371/journal.pone.0225704)
Supplement: S3 Appendix — (PDF) [file pone.0225704.s008.pdf]

Accounting for other effects

Secondary effects and relation

Density

Work-oriented (and thus colleagues) groups are more often present during working days, when the studied environment presents a lower density [1]. Tables 1 and 2 show the observable dependence at fixed density ranges ($0 \leq \rho < 0.05$ ped/m^2 and $0.1 \leq \rho < 0.15$ ped/m^2, respectively). The higher density range, which has a better statistical sample, confirms that the major trends exposed in the main text are present also at a fixed density, with similar effect sizes (δ-values) for V, r and y. The effect size on x is reduced (compare to Table 3 in the main text), as could be expected since this is the observable most affected by density [2]. As shown in Tables 3 and 4, that report p and δ-values, respectively, for all density ranges, the impact of relation on triads becomes stronger with density (the breaking up of the trends exposed in the main text for high densities is actually due to the fact that only 2 colleague triads ever walk at a density higher than 0.15 ped/m^2, and thus the lower lines of the table correspond to a comparison between friends and families). We may also notice that, as expected, density decreases average values in velocity and spatial observables, with the exception of y ([2, 3]).

Table 1: Observable dependence on relation for triads in the $0 \leq \rho < 0.05$ ped/m^2 range. Lengths in millimetres, times in seconds.

Relation	N_g^k	V	r	x	y
Colleagues	33	1212 ± 21 (σ=123)	644 ± 22 (σ=127)	1158 ± 49 (σ=279)	682 ± 56 (σ=324)
Families	44	1055 ± 23 (σ=150)	663 ± 28 (σ=184)	1067 ± 48 (σ=319)	743 ± 63 (σ=421)
Friends	45	1063 ± 25 (σ=169)	607 ± 22 (σ=146)	1139 ± 44 (σ=293)	581 ± 52 (σ=348)
$F_{2,119}$		12.1	1.43	1.02	2.11
p		$1.66 \cdot 10^{-5}$	0.243	0.362	0.126
R^2		0.169	0.0235	0.0169	0.0342
δ		1.13	0.338	0.301	0.419

Table 2: Observable dependence on relation for triads in the $0.1 \leq \rho < 0.15$ ped/m^2 range. Lengths in millimetres, times in seconds.

Relation	N_g^k	V	r	x	y
Colleagues	23	1171 ± 22 (σ=107)	591 ± 30 (σ=144)	989 ± 39 (σ=187)	661 ± 70 (σ=335)
Families	163	1000 ± 12 (σ=155)	629 ± 11 (σ=141)	866 ± 22 (σ=279)	764 ± 28 (σ=360)
Friends	116	1030 ± 13 (σ=140)	558 ± 10 (σ=109)	906 ± 21 (σ=228)	630 ± 28 (σ=299)
$F_{2,299}$		13.7	9.9	2.71	5.57
p		$1.98 \cdot 10^{-6}$	$6.88 \cdot 10^{-5}$	0.0683	0.00421
R^2		0.0841	0.0621	0.0178	0.0359
δ		1.14	0.547	0.459	0.4

Table 3: p-values for relation corresponding to velocity and distance observables at different density ranges. Lengths in millimetres, times in seconds.

Density	V	r	x	y
0-0.05 ped/m^2	$1.66 \cdot 10^{-5}$	0.243	0.362	0.126
0.05-0.1 ped/m^2	$< 10^{-8}$	0.0197	$8.98 \cdot 10^{-5}$	0.0134
0.1-0.15 ped/m^2	$1.98 \cdot 10^{-6}$	$6.88 \cdot 10^{-5}$	0.0683	0.00421
0.15-0.2 ped/m^2	0.265	$2.47 \cdot 10^{-5}$	0.245	0.0228
0.2-0.25 ped/m^2	0.693	0.0216	0.841	0.488
0.25-0.3 ped/m^2	0.812	0.143	0.972	0.271

Table 4: Effect size δ-values for relation corresponding to velocity and distance observables at different density ranges. Lengths in millimetres, times in seconds.

Density	V	r	x	y
0-0.05 ped/m^2	1.13	0.338	0.301	0.419
0.05-0.1 ped/m^2	1.1	0.378	0.721	0.376
0.1-0.15 ped/m^2	1.14	0.547	0.459	0.4
0.15-0.2 ped/m^2	1.21	0.818	1.43	0.958
0.2-0.25 ped/m^2	0.12	0.707	0.0609	0.211
0.25-0.3 ped/m^2	0.205	1.33	0.0298	1

Gender

We now compare the results regarding relation for groups of given gender (namely all females, two females and one male, two males and one female, all males) in Tables 5, 6, 7 and 8, respectively. Although statistical significance is often hindered by the reduced number of females in colleague triads, both the analysis of the male large statistical sample, and of the effect sizes in all genders, confirm the trends of the main text (with the exception of the large y value presented by triads with two males).

Table 5: Observable dependence on relation for 3 females triads. Lengths in millimetres, times in seconds.

Relation	N_g^k	V	r	x	y
Colleagues	3	1139 ± 55 (σ=95)	531 ± 52 (σ=90.3)	1219 ± 40 (σ=68.9)	298 ± 50 (σ=86.1)
Families	32	990 ± 26 (σ=147)	596 ± 29 (σ=163)	960 ± 56 (σ=319)	637 ± 79 (σ=449)
Friends	117	1035 ± 13 (σ=145)	547 ± 9.6 (σ=104)	963 ± 23 (σ=251)	593 ± 26 (σ=277)
$F_{2,149}$		2.09	2.2	1.36	1.55
p		0.128	0.115	0.261	0.215
R^2		0.0272	0.0287	0.0179	0.0204
δ		1.03	0.41	0.837	0.78

Table 6: Observable dependence on relation for mixed triads with two females. Lengths in millimetres, times in seconds.

Relation	N_g^k	V	r	x	y
Colleagues	5	1096 ± 35 (σ=79)	535 ± 23 (σ=51.7)	1092 ± 45 (σ=101)	409 ± 110 (σ=236)
Families	204	963 ± 9.4 (σ=134)	610 ± 10 (σ=144)	850 ± 17 (σ=238)	746 ± 27 (σ=391)
Friends	22	1032 ± 31 (σ=146)	618 ± 33 (σ=154)	1028 ± 75 (σ=353)	673 ± 69 (σ=322)
$F_{2,228}$		4.75	0.707	6.96	2.16
p		0.00953	0.494	0.00117	0.118
R^2		0.04	0.00617	0.0575	0.0186
δ		0.996	0.586	1.03	0.867

Table 7: Observable dependence on relation for mixed triads with two males. Lengths in millimetres, times in seconds.

Relation	N_g^k	V	r	x	y
Colleagues	10	1147 ± 47 (σ=147)	635 ± 29 (σ=92.7)	964 ± 55 (σ=173)	832 ± 78 (σ=247)
Families	142	1003 ± 14 (σ=163)	644 ± 14 (σ=163)	901 ± 23 (σ=278)	784 ± 30 (σ=362)
Friends	21	981 ± 32 (σ=147)	592 ± 28 (σ=128)	983 ± 66 (σ=302)	633 ± 67 (σ=305)
$F_{2,170}$		4.07	1	0.947	1.86
p		0.0187	0.368	0.39	0.16
R^2		0.0457	0.0117	0.011	0.0214
δ		1.13	0.327	0.29	0.691

Table 8: Observable dependence on relation for three males triads. Lengths in millimetres, times in seconds.

Relation	N_g^k	V	r	x	y
Colleagues	61	1209 ± 21 (σ=161)	634 ± 21 (σ=162)	1121 ± 38 (σ=298)	653 ± 50 (σ=391)
Families	18	1138 ± 53 (σ=225)	638 ± 35 (σ=150)	864 ± 49 (σ=206)	796 ± 78 (σ=333)
Friends	52	1072 ± 25 (σ=180)	568 ± 16 (σ=114)	954 ± 33 (σ=241)	592 ± 44 (σ=314)
$F_{2,128}$		8.1	3.35	8.97	2.17
p		0.000485	0.0382	0.000225	0.118
R^2		0.112	0.0497	0.123	0.0328
δ		0.807	0.565	0.918	0.639

Age

Tables 9 and 10 show the observable dependence at fixed minimum age ranges (30-39 and 40-49 years, respectively). The age distribution of colleagues has a limited overlapping with the other categories (in particular families), something that hinders the extent of this analysis. The shown ranges are the ones in which there is a better balance in sample sizes, although in general such samples are quite limited. Effect size analysis confirms anyway that the trends in the main text are present even when age is fixed, and in the 30-39 years range they are strong enough to support statistical significance. Tables 11 and 12 show p-values and effect sizes, although it has to be noticed that no colleague

triad was present in the 8-19 years range (the 0-8 years range presents only families while the 60-69 years range presents only friends and as a results p and δ are undefined).

Table 9: Observable dependence on relation for triads with minimum age in the 30-39 years range. Lengths in millimetres, times in seconds.

Relation	N_g^k	V	r	x	y
Colleagues	20	1218 ± 29 (σ=129)	631 ± 36 (σ=163)	1193 ± 71 (σ=318)	617 ± 87 (σ=391)
Families	18	1006 ± 40 (σ=170)	720 ± 35 (σ=150)	810 ± 57 (σ=242)	1024 ± 110 (σ=469)
Friends	14	982 ± 40 (σ=150)	544 ± 26 (σ=98.8)	863 ± 51 (σ=190)	676 ± 88 (σ=330)
$F_{2,49}$		12.9	5.63	11.1	5.02
p		$3.09 \cdot 10^{-5}$	0.00629	0.000108	0.0104
R^2		0.346	0.187	0.311	0.17
δ		1.71	1.35	1.35	0.947

Table 10: Observable dependence on relation for triads with minimum age in the 40-49 years range. Lengths in millimetres, times in seconds.

Relation	N_g^k	V	r	x	y
Colleagues	40	1200 ± 28 (σ=179)	627 ± 22 (σ=140)	1067 ± 45 (σ=285)	705 ± 61 (σ=386)
Families	20	927 ± 31 (σ=138)	642 ± 34 (σ=151)	886 ± 60 (σ=268)	754 ± 91 (σ=408)
Friends	12	1017 ± 34 (σ=116)	592 ± 27 (σ=93.3)	1001 ± 81 (σ=281)	662 ± 39 (σ=136)
$F_{2,69}$		20.4	0.474	2.69	0.245
p		$1.11 \cdot 10^{-7}$	0.624	0.0753	0.783
R^2		0.371	0.0136	0.0722	0.00706
δ		1.64	0.371	0.649	0.275

Table 11: p-values for relation in different minimum age ranges. Lengths in millimetres, times in seconds.

Minimum age	V	r	x	y
8-19 years	0.439	$1.41 \cdot 10^{-5}$	0.0675	$3.05 \cdot 10^{-5}$
20-29 years	0.00208	0.617	0.274	0.141
30-39 years	$3.09 \cdot 10^{-5}$	0.00629	0.000108	0.0104
40-49 years	$1.11 \cdot 10^{-7}$	0.624	0.0753	0.783
50-59 years	0.0275	0.644	0.0836	0.757
$\geq$ 70 years	0.172	0.721	0.216	0.0913

Height

Tables 13 and 14 show the observable dependence on relation at fixed minimum height ranges (150-160 and 160-170 cm, respectively), showing that the distributions are still different in a significant way, and that the major patterns exposed in the main text are confirmed. Tables 15 and 16 show respectively the p and δ-values for all height ranges (the $140 - 150$ cm range does not present colleagues).

Table 12: Effect size δ-values for relation in different minimum age ranges. Lengths in millimetres, times in seconds.

Minimum age	V	r	x	y
8-19 years	0.102	0.58	0.241	0.556
20-29 years	1.54	0.211	0.324	0.583
30-39 years	1.71	1.35	1.35	0.947
40-49 years	1.64	0.371	0.649	0.275
50-59 years	1.91	0.483	1.65	0.425
$\geq$ 70 years	1.01	0.252	0.904	1.31

Table 13: Observable dependence on relation for triads with minimum height in the 150-160 cm range. Lengths in millimetres, times in seconds.

Relation	N_g^k	V	r	x	y
Colleagues	8	1087 $\pm$ 48 (σ=137)	573 $\pm$ 33 (σ=92.3)	1040 $\pm$ 51 (σ=144)	560 $\pm$ 88 (σ=248)
Families	119	955 $\pm$ 13 (σ=144)	609 $\pm$ 13 (σ=139)	875 $\pm$ 21 (σ=234)	722 $\pm$ 34 (σ=371)
Friends	94	1037 $\pm$ 16 (σ=154)	556 $\pm$ 11 (σ=108)	973 $\pm$ 29 (σ=280)	592 $\pm$ 29 (σ=281)
$F_{2,218}$		9.51	4.68	4.77	4.35
p		0.000109	0.0102	0.00942	0.0141
R^2		0.0803	0.0412	0.0419	0.0384
δ		0.92	0.42	0.716	0.442

Table 14: Observable dependence on relation for dyads with minimum height in the 160-170 cm range. Lengths in millimetres, times in seconds.

Relation	N_g^k	V	r	x	y
Colleagues	43	1171 $\pm$ 23 (σ=149)	609 $\pm$ 22 (σ=145)	1008 $\pm$ 32 (σ=210)	710 $\pm$ 60 (σ=394)
Families	89	990 $\pm$ 15 (σ=137)	617 $\pm$ 16 (σ=152)	882 $\pm$ 26 (σ=246)	755 $\pm$ 40 (σ=379)
Friends	88	1041 $\pm$ 17 (σ=161)	572 $\pm$ 12 (σ=116)	975 $\pm$ 27 (σ=253)	610 $\pm$ 33 (σ=305)
$F_{2,217}$		21	2.51	5.09	3.71
p		$< 10^{-8}$	0.0833	0.00691	0.0259
R^2		0.162	0.0226	0.0448	0.0331
δ		1.28	0.33	0.539	0.419

Table 15: p-values for relation at different minimum height ranges. Lengths in millimetres, times in seconds.

Minimum height	V	r	x	y
140-150 cm	0.632	0.341	0.0503	0.864
150-160 cm	0.000109	0.0102	0.00942	0.0141
160-170 cm	$< 10^{-8}$	0.0833	0.00691	0.0259
170-180 cm	$1.77 \cdot 10^{-7}$	0.0238	$1.66 \cdot 10^{-5}$	0.199

Table 16: Effect size δ-values for relation at different minimum height ranges. Lengths in millimetres, times in seconds.

Minimum height	V	r	x	y
140-150 cm	0.256	0.517	1.08	0.0907
150-160 cm	0.92	0.42	0.716	0.442
160-170 cm	1.28	0.33	0.539	0.419
170-180 cm	2.18	0.807	1.55	0.785

Secondary effects and gender

Density

Tables 17 and 18 show the dependence of observables on gender at fixed density ranges ($0.05 \leq \rho < 0.1$ ped/m^2 and $0.15 \leq \rho < 0.2$ ped/m^2, respectively). The results confirm that the trends shown in the main text are present also at fixed density, although the variation in the x variable at higher density is limited (as noticed in the analysis of relation). Tables 19 and 20 show the density dependence of p and δ-values (sample sizes at low and high densities are reduced).

Table 17: Observable dependence on gender in the $0.05 \leq \rho < 0.1$ ped/m^2 density range. Lengths in millimetres, times in seconds.

Gender	N_g^k	V	r	x	y
Three females	82	1062 ± 15 (σ=134)	558 ± 13 (σ=115)	979 ± 29 (σ=266)	584 ± 34 (σ=310)
Two females	78	1009 ± 17 (σ=151)	595 ± 14 (σ=126)	922 ± 32 (σ=285)	682 ± 40 (σ=356)
Two males	42	1037 ± 25 (σ=161)	634 ± 25 (σ=162)	944 ± 42 (σ=272)	790 ± 58 (σ=378)
Three males	66	1171 ± 24 (σ=196)	602 ± 18 (σ=149)	1067 ± 37 (σ=303)	618 ± 48 (σ=389)
$F_{3,264}$		13	3.19	3.36	3.45
p		$6.08 \cdot 10^{-8}$	0.0243	0.0193	0.0171
R^2		0.129	0.0349	0.0368	0.0377
δ		0.936	0.574	0.493	0.616

Table 18: Observable dependence on gender in the $0.15 \leq \rho < 0.2$ ped/m^2 density range. Lengths in millimetres, times in seconds.

Gender	N_g^k	V	r	x	y
Three females	48	948 ± 17 (σ=121)	524 ± 14 (σ=94.3)	846 ± 38 (σ=261)	629 ± 39 (σ=268)
Two females	97	959 ± 12 (σ=120)	593 ± 15 (σ=144)	815 ± 20 (σ=198)	755 ± 40 (σ=391)
Two males	63	995 ± 17 (σ=138)	630 ± 17 (σ=131)	821 ± 30 (σ=241)	790 ± 41 (σ=325)
Three males	26	1029 ± 30 (σ=154)	560 ± 23 (σ=117)	843 ± 32 (σ=165)	638 ± 56 (σ=283)
$F_{3,230}$		3.15	6.56	0.264	2.82
p		0.0258	0.000282	0.851	0.0397
R^2		0.0395	0.0789	0.00343	0.0355
δ		0.601	0.912	0.14	0.533

Relation

Tables 21, 22 and 23 show the gender dependence of observables in, respectively, colleagues, families and friends. We may see that although all male triads are always faster regardless of relation, the effect may be reduced once gender differences are considered for fixed relation (this happens specifically in colleagues and friends). Similar considerations may be proposed also for spatial observables. For example, although all male triads move in a more ordered way (lower y) when all relations are considered, all male families present high y values.

Table 19: p-values for gender in different density ranges. Lengths in millimetres, times in seconds.

Density	V	r	x	y
0-0.05 ped/m^2	$1.34\cdot10^{-6}$	0.14	0.868	0.228
0.05-0.1 ped/m^2	$6.08\cdot10^{-8}$	0.0243	0.0193	0.0171
0.1-0.15 ped/m^2	0.00282	0.000761	0.211	0.00146
0.15-0.2 ped/m^2	0.0258	0.000282	0.851	0.0397
0.2-0.25 ped/m^2	0.953	0.135	0.788	0.751
0.25-0.3 ped/m^2	0.748	0.979	0.143	0.217

Table 20: Effect size δ-values for gender in different density ranges. Lengths in millimetres, times in seconds.

Density	V	r	x	y
0-0.05 ped/m^2	1.47	0.709	0.195	0.581
0.05-0.1 ped/m^2	0.936	0.574	0.493	0.616
0.1-0.15 ped/m^2	0.656	0.658	0.358	0.65
0.15-0.2 ped/m^2	0.601	0.912	0.14	0.533
0.2-0.25 ped/m^2	0.212	0.756	0.324	0.301
0.25-0.3 ped/m^2	0.71	0.197	1.7	1.77

Table 21: Observable dependence on gender for colleagues. Lengths in millimetres, times in seconds.

Gender	N_g^k	V	r	x	y
Three females	3	1139 ± 55 (σ=95)	531 ± 52 (σ=90.3)	1219 ± 40 (σ=68.9)	298 ± 50 (σ=86.1)
Two females	5	1096 ± 35 (σ=79)	535 ± 23 (σ=51.7)	1092 ± 45 (σ=101)	409 ± 110 (σ=236)
Two males	10	1147 ± 47 (σ=147)	635 ± 29 (σ=92.7)	964 ± 55 (σ=173)	832 ± 78 (σ=247)
Three males	61	1209 ± 21 (σ=161)	634 ± 21 (σ=162)	1121 ± 38 (σ=298)	653 ± 50 (σ=391)
$F_{3,75}$		1.25	1.05	1.11	2.42
p		0.299	0.376	0.352	0.0724
R^2		0.0475	0.0403	0.0424	0.0884
δ		0.721	1.13	1.61	2.36

Age

Tables 24 and 25 show the dependence of observables on gender at fixed minimum age ranges (8-19 years and 30-39 years, respectively). The patterns exposed in the main text are fundamentally present also when age is fixed, at least in these two age ranges. In all the ranges examined, same gender male groups are faster, although effect size may vary. The spatial observable patterns are confirmed in the tables below, but may vary in other ranges (e.g., as observed above, groups with three males in the 0-7 years old range present very high y values). Tables 26 and 27 show the p and δ-values for gender in different minimum age ranges.

Table 22: Observable dependence on gender for families. Lengths in millimetres, times in seconds.

Gender	N_g^k	V	r	x	y
Three females	32	990 ± 26 (σ=147)	596 ± 29 (σ=163)	960 ± 56 (σ=319)	637 ± 79 (σ=449)
Two females	204	963 ± 9.4 (σ=134)	610 ± 10 (σ=144)	850 ± 17 (σ=238)	746 ± 27 (σ=391)
Two males	142	1003 ± 14 (σ=163)	644 ± 14 (σ=163)	901 ± 23 (σ=278)	784 ± 30 (σ=362)
Three males	18	1138 ± 53 (σ=225)	638 ± 35 (σ=150)	864 ± 49 (σ=206)	796 ± 78 (σ=333)
$F_{3,392}$		8.19	1.7	2.26	1.37
p		$2.69 \cdot 10^{-5}$	0.167	0.0814	0.253
R^2		0.059	0.0128	0.017	0.0103
δ		1.22	0.293	0.44	0.386

Table 23: Observable dependence on gender for friends. Lengths in millimetres, times in seconds.

Gender	N_g^k	V	r	x	y
Three females	117	1035 ± 13 (σ=145)	547 ± 9.6 (σ=104)	963 ± 23 (σ=251)	593 ± 26 (σ=277)
Two females	22	1032 ± 31 (σ=146)	618 ± 33 (σ=154)	1028 ± 75 (σ=353)	673 ± 69 (σ=322)
Two males	21	981 ± 32 (σ=147)	592 ± 28 (σ=128)	983 ± 66 (σ=302)	633 ± 67 (σ=305)
Three males	52	1072 ± 25 (σ=180)	568 ± 16 (σ=114)	954 ± 33 (σ=241)	592 ± 44 (σ=314)
$F_{3,208}$		1.79	2.87	0.439	0.536
p		0.151	0.0373	0.726	0.658
R^2		0.0251	0.0398	0.00628	0.00767
δ		0.532	0.633	0.264	0.254

Table 24: Observable dependence on gender in the 8-19 years minimum age range. Lengths in millimetres, times in seconds.

Gender	N_g^k	V	r	x	y
Three females	66	1013 ± 18 (σ=147)	530 ± 13 (σ=105)	958 ± 35 (σ=287)	545 ± 35 (σ=285)
Two females	99	982 ± 14 (σ=135)	600 ± 14 (σ=136)	836 ± 21 (σ=205)	739 ± 36 (σ=357)
Two males	82	1034 ± 16 (σ=148)	631 ± 17 (σ=152)	932 ± 33 (σ=295)	755 ± 38 (σ=343)
Three males	23	1090 ± 40 (σ=192)	561 ± 26 (σ=123)	916 ± 37 (σ=177)	599 ± 63 (σ=302)
$F_{3,266}$		4.06	7.51	3.6	6.48
p		0.00758	$7.63 \cdot 10^{-5}$	0.0141	0.000302
R^2		0.0438	0.0781	0.039	0.0681
δ		0.738	0.763	0.502	0.662

Height

Tables 28 and 29 show the dependence of observables on gender at fixed minimum height ranges ($\leq$ 140 and 160-170 cm, respectively). The results show that gender related differences are still present when we consider individuals of similar height, in particular concerning velocity (for spatial observables, the discussion reported above for the high y values of male triads with children obviously affects also the results concerning the lower minimum height ranges). p and δ-values are reported in Tables 30 and 31.

Table 25: Observable dependence on gender in the 30-39 years minimum age range. Lengths in millimetres, times in seconds.

Gender	N_g^k	V	r	x	y
Three females	11	1010 ± 49 (σ=161)	614 ± 59 (σ=196)	943 ± 66 (σ=220)	761 ± 160 (σ=536)
Two females	11	1054 ± 55 (σ=182)	655 ± 48 (σ=160)	807 ± 66 (σ=220)	882 ± 160 (σ=533)
Two males	10	1005 ± 54 (σ=172)	652 ± 27 (σ=85.2)	791 ± 52 (σ=165)	879 ± 75 (σ=236)
Three males	20	1173 ± 37 (σ=166)	636 ± 36 (σ=163)	1169 ± 78 (σ=349)	669 ± 86 (σ=386)
$F_{3,48}$		3.08	0.141	5.98	0.751
p		0.0362	0.935	0.0015	0.527
R^2		0.161	0.00874	0.272	0.0448
δ		0.997	0.23	1.25	0.482

Table 26: p-values for gender in different minimum age ranges. Lengths in millimetres, times in seconds.

Minimum age	V	r	x	y
0-7 years	0.00539	0.144	0.872	0.128
8-19 years	0.00758	$7.63{\cdot}10^{-5}$	0.0141	0.000302
20-29 years	0.0512	0.505	0.796	0.203
30-39 years	0.0362	0.935	0.0015	0.527
40-49 years	$2.39{\cdot}10^{-6}$	0.563	0.181	0.462
50-59 years	0.663	0.51	0.838	0.545
$\geq$ 70 years	0.427	0.046	0.827	0.1

Table 27: Effect size δ-values for gender in different minimum age ranges. Lengths in millimetres, times in seconds.

Minimum age	V	r	x	y
0-7 years	1.36	1.75	0.419	2.04
8-19 years	0.738	0.763	0.502	0.662
20-29 years	0.608	0.382	0.281	0.636
30-39 years	0.997	0.23	1.25	0.482
40-49 years	1.69	0.546	0.722	0.538
50-59 years	0.986	0.727	0.58	0.96
$\geq$ 70 years	2.11	2.8	0.617	2.33

Table 28: Observable dependence on gender in the $\leq$ 140 cm minimum height range. Lengths in millimetres, times in seconds.

Gender	N_g^k	V	r	x	y
Three females	10	1003 ± 41 (σ=129)	546 ± 39 (σ=123)	998 ± 110 (σ=334)	452 ± 39 (σ=123)
Two females	63	977 ± 17 (σ=138)	630 ± 19 (σ=149)	844 ± 37 (σ=293)	774 ± 49 (σ=390)
Two males	51	993 ± 26 (σ=189)	673 ± 25 (σ=175)	918 ± 41 (σ=291)	793 ± 53 (σ=379)
Three males	10	1212 ± 69 (σ=217)	678 ± 52 (σ=166)	968 ± 57 (σ=181)	751 ± 100 (σ=331)
$F_{3,130}$		5.75	2.08	1.36	2.42
p		0.001	0.107	0.258	0.0691
R^2		0.117	0.0457	0.0305	0.0529
δ		1.57	0.902	0.516	0.968

Table 29: Observable dependence on gender in the 160-170 cm minimum height range. Lengths in millimetres, times in seconds.

Gender	N_g^k	V	r	x	y
Three females	54	1020 ± 19 (σ=143)	553 ± 18 (σ=132)	978 ± 31 (σ=231)	589 ± 48 (σ=354)
Two females	55	988 ± 18 (σ=132)	599 ± 18 (σ=132)	917 ± 33 (σ=246)	703 ± 51 (σ=378)
Two males	50	1027 ± 20 (σ=140)	633 ± 19 (σ=137)	894 ± 36 (σ=257)	777 ± 44 (σ=310)
Three males	61	1137 ± 24 (σ=185)	605 ± 18 (σ=141)	978 ± 32 (σ=247)	690 ± 47 (σ=368)
$F_{3,216}$		10.5	3.06	1.64	2.45
p		$1.86 \cdot 10^{-6}$	0.0292	0.18	0.0649
R^2		0.127	0.0408	0.0223	0.0329
δ		0.92	0.592	0.348	0.565

Table 30: p-values for gender in different minimum height ranges. Lengths in millimetres, times in seconds.

Minimum height	V	r	x	y
< 140 cm	0.001	0.107	0.258	0.0691
140-150 cm	0.0149	0.493	0.497	0.219
150-160 cm	0.00367	0.133	0.145	0.141
160-170 cm	$1.86 \cdot 10^{-6}$	0.0292	0.18	0.0649
170-180 cm	0.0503	0.3	0.0851	0.0278

Table 31: Effect size δ-values for gender in different minimum height ranges. Lengths in millimetres, times in seconds.

Minimum height	V	r	x	y
< 140 cm	1.57	0.902	0.516	0.968
140-150 cm	1.34	1.52	0.546	1.06
150-160 cm	0.806	0.552	0.436	0.693
160-170 cm	0.92	0.592	0.348	0.565
170-180 cm	1.2	1.54	0.902	1.12

References

[1] Brščić, Dražen and Zanlungo, Francesco and Kanda, Takayuki, *Density and velocity patterns during one year of pedestrian tracking*, Transportation Research Procedia, 2, 77–86, 2014

[2] Zanlungo F, and Kanda T, *A mesoscopic model for the effect of density on pedestrian group dynamics*, EPL (Europhysics Letters), 2015; 111, 38007.

[3] Zanlungo F, Brščić D and Kanda T, *Spatial-size scaling of pedestrian groups under growing density conditions* Physical Review E 91 (6), 062810 (2015)
