# Supplementary material for: Intrinsic group behaviour II: On the dependence of triad spatial dynamics on social and personal features; and on the effect of social interaction on small group dynamics
Source: PLoS One. 2019 Dec 3;14(12):e0225704. doi: 10.1371/journal.pone.0225704 (PMC6890182; doi:10.1371/journal.pone.0225704)
Supplement: S4 Appendix — (PDF) [file pone.0225704.s009.pdf]

# Statistical analysis of overall probability distributions

## Purpose

Table 1 provides a statistical analysis of the overall probability distributions for the purpose categories in triads.

Table 1: Statistical analysis of the overall probability distributions for the purpose categories in triads. Lengths in millimetres, times in seconds.

| Purpose | $N^k$ | $V$ | $r$ | $x$ | $y$ |
|---|---|---|---|---|---|
| Leisure | 26120 | 998 ± 1.3 ($\sigma$=206) | 594 ± 1.1 ($\sigma$=172) | 914 ± 2.2 ($\sigma$=360) | 688 ± 3 ($\sigma$=479) |
| Work | 3375 | 1191 ± 3.6 ($\sigma$=211) | 599 ± 2.7 ($\sigma$=155) | 1112 ± 5.8 ($\sigma$=337) | 596 ± 7.2 ($\sigma$=417) |
| $F_{1,29493}$ | | 2590 | 3.31 | 922 | 115 |
| $p$ | | $< 10^{-8}$ | 0.0687 | $< 10^{-8}$ | $< 10^{-8}$ |
| $R^2$ | | 0.0809 | 0.000112 | 0.0303 | 0.00388 |
| $\delta$ | | 0.932 | 0.0333 | 0.555 | 0.196 |

## Relation

Table 2 provides a statistical analysis of the overall probability distributions for the relation categories in triads.

Table 2: Statistical analysis of the overall probability distributions for the relation categories in triads. Lengths in millimetres, times in seconds.

| Relation | $N^k$ | $V$ | $r$ | $x$ | $y$ |
|---|---|---|---|---|---|
| Colleagues | 3370 | 1189 ± 3.6 ($\sigma$=210) | 602 ± 2.7 ($\sigma$=156) | 1108 ± 5.9 ($\sigma$=340) | 606 ± 7.3 ($\sigma$=422) |
| Families | 15599 | 981 ± 1.6 ($\sigma$=206) | 619 ± 1.5 ($\sigma$=188) | 876 ± 2.9 ($\sigma$=366) | 750 ± 4.1 ($\sigma$=518) |
| Friends | 10526 | 1024 ± 2 ($\sigma$=204) | 555 ± 1.3 ($\sigma$=137) | 972 ± 3.3 ($\sigma$=343) | 594 ± 3.9 ($\sigma$=396) |
| $F_{2,29492}$ | | 1410 | 460 | 673 | 399 |
| $p$ | | $< 10^{-8}$ | $< 10^{-8}$ | $< 10^{-8}$ | $< 10^{-8}$ |
| $R^2$ | | 0.0875 | 0.0302 | 0.0436 | 0.0263 |
| $\delta$ | | 1 | 0.378 | 0.641 | 0.331 |

## Gender

Table 3 provides a statistical analysis of the overall probability distributions for the gender categories in triads.

## Age

Table 4 provides a statistical analysis of the overall probability distributions for the minimum age ranges in triads.

Table 3: Statistical analysis of the overall probability distributions for the gender categories in triads. Lengths in millimetres, times in seconds.

| Gender | $N^k$ | $V$ | $r$ | $x$ | $y$ |
|---|---|---|---|---|---|
| Three females | 7826 | 1013 ± 2.3 ($\sigma$=200) | 557 ± 1.7 ($\sigma$=147) | 965 ± 3.9 ($\sigma$=346) | 607 ± 4.7 ($\sigma$=418) |
| Two females | 9689 | 976 ± 2 ($\sigma$=199) | 601 ± 1.8 ($\sigma$=177) | 886 ± 3.6 ($\sigma$=354) | 705 ± 5 ($\sigma$=494) |
| Two males | 6460 | 998 ± 2.6 ($\sigma$=207) | 631 ± 2.3 ($\sigma$=183) | 900 ± 4.8 ($\sigma$=383) | 770 ± 6.2 ($\sigma$=499) |
| Three males | 5520 | 1134 ± 3.2 ($\sigma$=234) | 593 ± 2.2 ($\sigma$=163) | 1028 ± 4.8 ($\sigma$=355) | 622 ± 6.1 ($\sigma$=455) |
| $F_{3,29491}$ | | 730 | 234 | 223 | 179 |
| $p$ | | $< 10^{-8}$ | $< 10^{-8}$ | $< 10^{-8}$ | $< 10^{-8}$ |
| $R^2$ | | 0.0692 | 0.0233 | 0.0222 | 0.0178 |
| $\delta$ | | 0.747 | 0.45 | 0.402 | 0.355 |

Table 4: Statistical analysis of the overall probability distributions for the minimum age ranges in triads. Lengths in millimetres, times in seconds.

| Minimum age | $N^k$ | $V$ | $r$ | $x$ | $y$ |
|---|---|---|---|---|---|
| 0-7 years | 4330 | 962 ± 3.2 ($\sigma$=210) | 614 ± 3 ($\sigma$=200) | 870 ± 5.6 ($\sigma$=366) | 732 ± 8 ($\sigma$=527) |
| 8-19 years | 11873 | 1010 ± 1.9 ($\sigma$=207) | 581 ± 1.5 ($\sigma$=167) | 899 ± 3.2 ($\sigma$=352) | 673 ± 4.3 ($\sigma$=468) |
| 20-29 years | 5663 | 1030 ± 2.7 ($\sigma$=204) | 575 ± 2 ($\sigma$=151) | 972 ± 4.5 ($\sigma$=338) | 637 ± 5.7 ($\sigma$=425) |
| 30-39 years | 2497 | 1062 ± 4.5 ($\sigma$=223) | 623 ± 3.6 ($\sigma$=179) | 978 ± 7.6 ($\sigma$=379) | 718 ± 10 ($\sigma$=511) |
| 40-49 years | 3226 | 1086 ± 4.2 ($\sigma$=238) | 610 ± 2.8 ($\sigma$=157) | 1010 ± 6.5 ($\sigma$=371) | 673 ± 7.8 ($\sigma$=442) |
| 50-59 years | 1253 | 1059 ± 6 ($\sigma$=214) | 598 ± 4.3 ($\sigma$=151) | 1054 ± 10 ($\sigma$=360) | 572 ± 12 ($\sigma$=437) |
| 60-69 years | 222 | 944 ± 19 ($\sigma$=285) | 675 ± 9.5 ($\sigma$=141) | 1311 ± 25 ($\sigma$=377) | 585 ± 33 ($\sigma$=496) |
| ≥ 70 years | 431 | 931 ± 7.9 ($\sigma$=165) | 690 ± 9.5 ($\sigma$=197) | 854 ± 19 ($\sigma$=400) | 969 ± 26 ($\sigma$=540) |
| $F_{7,29487}$ | | 130 | 70.2 | 130 | 51.3 |
| $p$ | | $< 10^{-8}$ | $< 10^{-8}$ | $< 10^{-8}$ | $< 10^{-8}$ |
| $R^2$ | | 0.0299 | 0.0164 | 0.03 | 0.012 |
| $\delta$ | | 0.668 | 0.742 | 1.16 | 0.854 |

## Height

Table 5 provides a statistical analysis of the overall probability distributions for the minimum height ranges in triads.

Table 5: Statistical analysis of the overall probability distributions for the minimum height ranges in triads. Lengths in millimetres, times in seconds.

| Minimum height | $N^k$ | $V$ | $r$ | $x$ | $y$ |
|---|---|---|---|---|---|
| < 140 cm | 4479 | 990 ± 3.3 ($\sigma$=219) | 641 ± 3 ($\sigma$=203) | 881 ± 5.9 ($\sigma$=393) | 767 ± 8 ($\sigma$=536) |
| 140-150 cm | 1897 | 1032 ± 4.9 ($\sigma$=212) | 595 ± 4.2 ($\sigma$=181) | 871 ± 8.2 ($\sigma$=356) | 769 ± 12 ($\sigma$=520) |
| 150-160 cm | 10195 | 991 ± 2 ($\sigma$=199) | 588 ± 1.6 ($\sigma$=157) | 918 ± 3.5 ($\sigma$=351) | 673 ± 4.5 ($\sigma$=456) |
| 160-170 cm | 10153 | 1033 ± 2.1 ($\sigma$=214) | 581 ± 1.6 ($\sigma$=164) | 962 ± 3.4 ($\sigma$=342) | 645 ± 4.5 ($\sigma$=452) |
| 170-180 cm | 2741 | 1117 ± 4.6 ($\sigma$=241) | 594 ± 3.1 ($\sigma$=161) | 1052 ± 7.6 ($\sigma$=396) | 607 ± 8.5 ($\sigma$=443) |
| $F_{5,29489}$ | | 186 | 87.1 | 107 | 68.9 |
| $p$ | | $< 10^{-8}$ | $< 10^{-8}$ | $< 10^{-8}$ | $< 10^{-8}$ |
| $R^2$ | | 0.0305 | 0.0145 | 0.0178 | 0.0115 |
| $\delta$ | | 1.3 | 0.904 | 0.546 | 0.325 |
