# Supplementary material for: Intrinsic group behaviour II: On the dependence of triad spatial dynamics on social and personal features; and on the effect of social interaction on small group dynamics
Source: PLoS One. 2019 Dec 3;14(12):e0225704. doi: 10.1371/journal.pone.0225704 (PMC6890182; doi:10.1371/journal.pone.0225704)
Supplement: S5 Appendix — (PDF) [file pone.0225704.s010.pdf]

Interaction and intrinsic properties

The effect of interaction on dyads

Tables 1 and 2 show the dependence on, respectively, relation and gender, of the dynamics of socially interacting dyads, while Tables 3 and 4 report the corresponding non socially interacting data. It can be easily noticed that socially interacting groups present the same characteristics observed before distinguishing for interaction, with strong effect sizes and significant differences in all observables. For non-interacting groups we may notice that, although differences are still present and strong in the V, x and y observable, the statistical significance of the dependence of the absolute distance r on both gender and relation is lost with lack of interaction. Nevertheless, since the effect sizes are quite similar in the interacting and non-interacting cases, this result is probably just due to the reduced sample of non-interacting dyads.

Table 1: Observable dependence on relation for socially interacting dyads. Lengths in millimetres, times in seconds.

Relation	N_g^k	V	r	x	y
Colleagues	256	1257 ± 9.9 (σ= 158)	821 ± 12 (σ= 200)	700 ± 8.3 (σ= 133)	312 ± 17 (σ= 265)
Couples	88	1084 ± 18 (σ= 166)	683 ± 19 (σ= 176)	577 ± 10 (σ= 95.1)	277 ± 23 (σ= 214)
Families	193	1081 ± 14 (σ= 188)	823 ± 21 (σ= 288)	585 ± 11 (σ= 159)	446 ± 26 (σ= 365)
Friends	274	1125 ± 12 (σ= 201)	771 ± 10 (σ= 172)	666 ± 7.6 (σ= 126)	281 ± 14 (σ= 226)
$F_{3,807}$		44.1	11.3	36.7	15.6
p		$< 10^{-8}$	$2.97 \cdot 10^{-7}$	$< 10^{-8}$	$< 10^{-8}$
R^2		0.141	0.0402	0.12	0.0548
δ		1.02	0.539	0.987	0.519

Table 2: Observable dependence on gender for socially interacting dyads. Lengths in millimetres, times in seconds.

Gender	N_g^k	V	r	x	y
Two females	222	1093 ± 13 (σ= 195)	770 ± 14 (σ= 208)	644 ± 8.1 (σ= 121)	295 ± 20 (σ= 291)
Mixed	300	1094 ± 10 (σ= 174)	785 ± 14 (σ= 250)	609 ± 8.8 (σ= 152)	372 ± 18 (σ= 311)
Two males	348	1234 ± 9.6 (σ= 179)	819 ± 11 (σ= 202)	684 ± 7.6 (σ= 142)	328 ± 15 (σ= 272)
$F_{2,867}$		62.4	3.67	23	4.6
p		$< 10^{-8}$	0.0258	$< 10^{-8}$	0.0103
R^2		0.126	0.0084	0.0503	0.0105
δ		0.761	0.238	0.512	0.254

Tables 5, 6, 7, 8, 9, 10 and 11 show the effect of interaction on, respectively, colleagues, couples, friends, families, two male, two female and mixed-gender dyads. It may be noticed that interaction always affects velocity (interacting dyads are slower), distance (interacting dyads are closer), and depth (interacting dyads have less depth, i.e., move more abreast). In couples, colleagues and two males it also affects abreast distance x (shorter for interacting dyads; in families and friends we have the opposite

Table 3: Observable dependence on relation for non-socially interacting dyads. Lengths in millimetres, times in seconds.

Relation	N_g^k	V	r	x	y
Colleagues	102	1316 ± 14 (σ= 146)	927 ± 28 (σ= 282)	763 ± 20 (σ= 200)	387 ± 34 (σ= 345)
Couples	8	1264 ± 34 (σ= 97.4)	1053 ± 120 (σ= 327)	854 ± 110 (σ= 316)	445 ± 120 (σ= 335)
Families	53	1139 ± 30 (σ= 221)	1010 ± 42 (σ= 307)	575 ± 29 (σ= 210)	690 ± 58 (σ= 422)
Friends	44	1217 ± 27 (σ= 178)	924 ± 43 (σ= 283)	636 ± 26 (σ= 172)	520 ± 59 (σ= 393)
$F_{3,203}$		12.4	1.37	12.7	7.54
p		$1.8{\cdot}10^{-7}$	0.253	$1.29{\cdot}10^{-7}$	$8.3{\cdot}10^{-5}$
R^2		0.155	0.0198	0.158	0.1
δ		1.01	0.444	1.24	0.814

Table 4: Observable dependence on gender for non-socially interacting dyads. Lengths in millimetres, times in seconds.

Gender	N_g^k	V	r	x	y
Two females	30	1170 ± 29 (σ= 159)	936 ± 54 (σ= 298)	673 ± 25 (σ= 136)	514 ± 70 (σ= 381)
Mixed	71	1185 ± 24 (σ= 198)	985 ± 36 (σ= 307)	626 ± 29 (σ= 246)	600 ± 52 (σ= 436)
Two males	118	1311 ± 15 (σ= 164)	928 ± 26 (σ= 278)	742 ± 20 (σ= 216)	409 ± 31 (σ= 341)
$F_{2,216}$		15	0.886	6.41	5.59
p		$7.66{\cdot}10^{-7}$	0.414	0.00197	0.00431
R^2		0.122	0.00814	0.056	0.0492
δ		0.868	0.199	0.51	0.502

effect but with a very weak effect size). Comparison of pdf is provided, in the two male dyad case, in Figures 1, 2, 3, 4, for, respectively, V, r, x and y. We chose to use the two male case due to its large sample, although some other categories present much stronger effect sizes on interaction (e.g., couples).

Table 5: Observable dependence on interaction for colleague dyads. Lengths in millimetres, times in seconds.

Colleagues	N_g^k	V	r	x	y
Interacting	256	1257 ± 9.9 (σ= 158)	821 ± 13 (σ= 200)	700 ± 8.3 (σ= 133)	312 ± 17 (σ= 265)
Non-interacting	102	1316 ± 15 (σ= 146)	927 ± 28 (σ= 282)	763 ± 20 (σ= 200)	387 ± 34 (σ= 345)
$F_{1,356}$		10.6	15.9	12	4.85
p		0.00127	$8.11{\cdot}10^{-5}$	0.000606	0.0283
R^2		0.0288	0.0427	0.0325	0.0134
δ		0.381	0.468	0.407	0.259

Table 6: Observable dependence on interaction for couples. Lengths in millimetres, times in seconds.

Couples	N_g^k	V	r	x	y
Interacting	88	1084 ± 18 (σ= 166)	683 ± 19 (σ= 176)	577 ± 10 (σ= 95.1)	277 ± 23 (σ= 214)
Non-interacting	8	1264 ± 37 (σ= 97.4)	1053 ± 120 (σ= 327)	854 ± 120 (σ= 316)	445 ± 130 (σ= 335)
$F_{1,94}$		8.93	26.4	33.2	3.95
p		0.00358	$1.53 \cdot 10^{-6}$	$1.06 \cdot 10^{-7}$	0.0498
R^2		0.0868	0.219	0.261	0.0403
δ		1.11	1.93	2.2	0.746

Table 7: Observable dependence on interaction for friend dyads. Lengths in millimetres, times in seconds.

Friends	N_g^k	V	r	x	y
Interacting	274	1125 ± 12 (σ= 201)	771 ± 10 (σ= 172)	666 ± 7.6 (σ= 126)	281 ± 14 (σ= 226)
Non-interacting	44	1217 ± 27 (σ= 178)	924 ± 43 (σ= 283)	636 ± 26 (σ= 172)	520 ± 60 (σ= 393)
$F_{1,316}$		8.14	24.1	1.91	32.9
p		0.00463	$1.46 \cdot 10^{-6}$	0.168	$2.25 \cdot 10^{-8}$
R^2		0.0251	0.0709	0.006	0.0943
δ		0.465	0.801	0.225	0.936

Table 8: Observable dependence on interaction for family dyads. Lengths in millimetres, times in seconds.

Families	N_g^k	V	r	x	y
Interacting	193	1081 ± 14 (σ= 188)	823 ± 21 (σ= 288)	585 ± 11 (σ= 159)	446 ± 26 (σ= 365)
Non-interacting	53	1139 ± 31 (σ= 221)	1010 ± 43 (σ= 307)	575 ± 29 (σ= 210)	690 ± 59 (σ= 422)
$F_{1,244}$		3.63	16.9	0.141	17.2
p		0.058	$5.41 \cdot 10^{-5}$	0.708	$4.68 \cdot 10^{-5}$
R^2		0.0146	0.0647	0.000576	0.0658
δ		0.297	0.64	0.0584	0.646

Table 9: Observable dependence on interaction for two male dyads. Lengths in millimetres, times in seconds.

Two males	N_g^k	V	r	x	y
Interacting	348	1234 ± 9.6 (σ= 179)	819 ± 11 (σ= 202)	684 ± 7.6 (σ= 142)	328 ± 15 (σ= 272)
Non-interacting	118	1311 ± 15 (σ= 164)	928 ± 26 (σ= 278)	742 ± 20 (σ= 216)	409 ± 32 (σ= 341)
$F_{1,464}$		16.9	20.8	11	6.8
p		$4.6 \cdot 10^{-5}$	$6.43 \cdot 10^{-6}$	0.000991	0.00942
R^2		0.0352	0.043	0.0231	0.0144
δ		0.439	0.487	0.354	0.278

Table 10: Observable dependence on interaction for two female dyads. Lengths in millimetres, times in seconds.

Two Females	N_g^k	V	r	x	y
Interacting	222	1093 ± 13 (σ= 195)	770 ± 14 (σ= 208)	644 ± 8.1 (σ= 121)	295 ± 20 (σ= 291)
Non-interacting	30	1170 ± 30 (σ= 159)	936 ± 55 (σ= 298)	673 ± 25 (σ= 136)	514 ± 71 (σ= 381)
$F_{1,250}$		4.26	14.8	1.46	13.7
p		0.0401	0.000149	0.228	0.000266
R^2		0.0167	0.056	0.00581	0.0519
δ		0.403	0.753	0.236	0.723

Table 11: Observable dependence on interaction for mixed gender dyads. Lengths in millimetres, times in seconds.

Mixed	N_g^k	V	r	x	y
Interacting	300	1094 ± 10 (σ= 174)	785 ± 14 (σ= 250)	609 ± 8.8 (σ= 152)	372 ± 18 (σ= 311)
Non-interacting	71	1185 ± 24 (σ= 198)	985 ± 37 (σ= 307)	626 ± 29 (σ= 246)	600 ± 52 (σ= 436)
$F_{1,369}$		14.8	33.3	0.545	25.9
p		0.000142	$1.67 \cdot 10^{-8}$	0.461	$5.73 \cdot 10^{-7}$
R^2		0.0385	0.0828	0.00148	0.0656
δ		0.509	0.764	0.0978	0.674

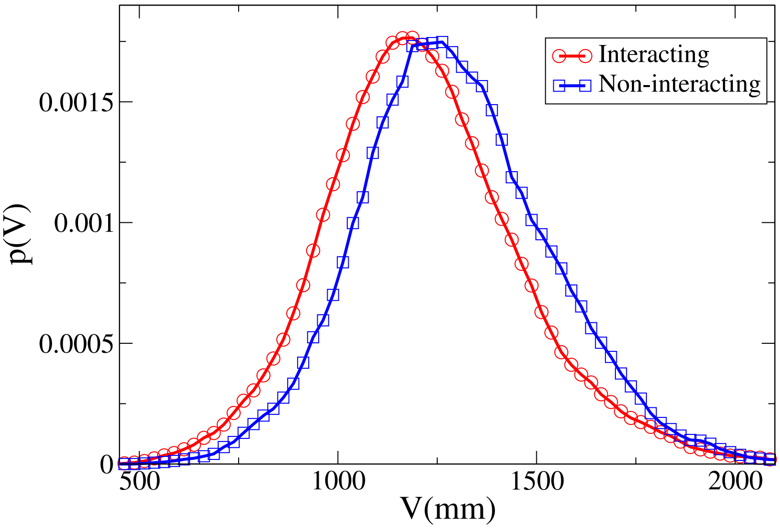

Figure 1: Pdf of the V observable in dyads with two males: interacting (red, circles) and non-interacting (blue, squares).

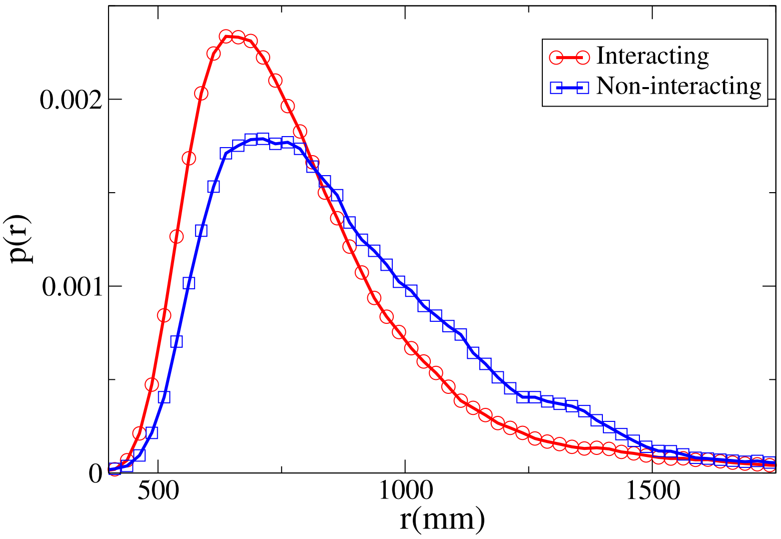

Figure 2: Pdf of the r observable in dyads with two males: interacting (red, circles) and non-interacting (blue, squares).

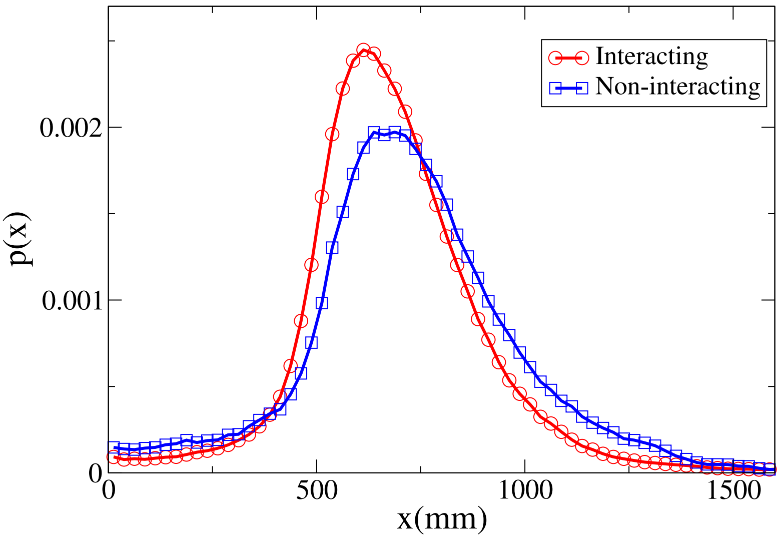

Figure 3: Pdf of the x observable in dyads with two males: interacting (red, circles) and non-interacting (blue, squares).

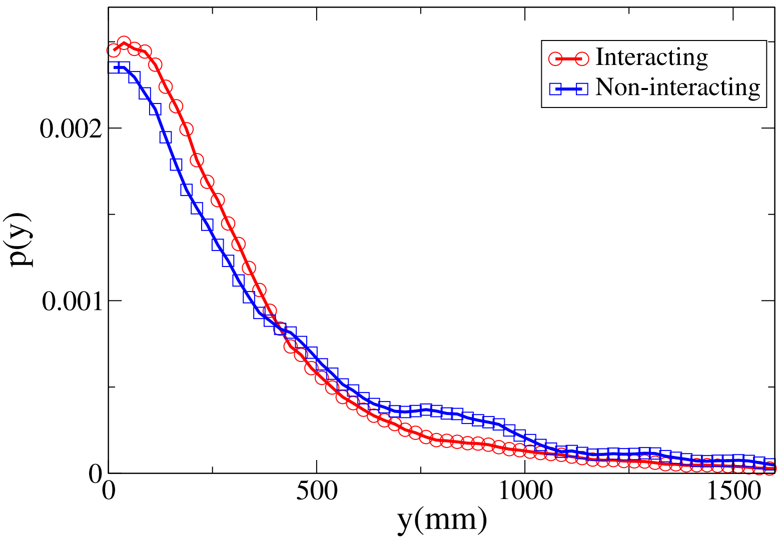

Figure 4: Pdf of the y observable in dyads with two males: interacting (red, circles) and non-interacting (blue, squares).

The effect of interaction on triads

Tables 12 and 13 show the dependence on, respectively relation and gender of the dynamics of socially interacting triads, while Tables 14 and 15 report the corresponding non socially interacting data. Again, it may be noticed that socially interacting groups present the same characteristics observed before distinguishing for interaction, with strong effect sizes and significant differences in all observables. For non-interacting groups we may say that statistical significance is not very strong for the gender dependence of distance observables. Anyway, as in the dyadic case, effect sizes are similar regardless of interaction, suggesting that the lack of statistical significance for these observables may be due to the limited sample size.

Table 12: Observable dependence on relation for socially interacting triads. Lengths in millimetres, times in seconds.

Relation	N_g^k	V	r	x	y
Colleagues	52	1168 ± 19 (σ=139)	577 ± 16 (σ=112)	1156 ± 36 (σ=262)	510 ± 43 (σ=310)
Families	322	981 ± 8.5 (σ=153)	607 ± 8.3 (σ=150)	886 ± 14 (σ=252)	707 ± 21 (σ=380)
Friends	162	1032 ± 12 (σ=153)	552 ± 9.2 (σ=117)	990 ± 21 (σ=271)	563 ± 21 (σ=268)
$F_{2,533}$		35.3	8.84	28.1	13.8
p		$< 10^{-8}$	0.000167	$< 10^{-8}$	$1.39 \cdot 10^{-6}$
R^2		0.117	0.0321	0.0954	0.0493
δ		1.24	0.395	1.07	0.53

Table 13: Observable dependence on gender for socially interacting triads. Lengths in millimetres, times in seconds.

Gender	N_g^k	V	r	x	y
Three females	129	1024 ± 13 (σ=145)	550 ± 11 (σ=122)	979 ± 24 (σ=272)	566 ± 28 (σ=314)
Two females	182	962 ± 10 (σ=134)	593 ± 11 (σ=143)	887 ± 19 (σ=254)	682 ± 28 (σ=374)
Two males	135	992 ± 13 (σ=151)	623 ± 13 (σ=153)	917 ± 23 (σ=263)	718 ± 30 (σ=350)
Three males	90	1141 ± 19 (σ=180)	575 ± 12 (σ=118)	1046 ± 30 (σ=283)	570 ± 34 (σ=321)
$F_{3,532}$		30.1	6.67	8.34	6.35
p		$< 10^{-8}$	0.000199	$1.98 \cdot 10^{-5}$	0.000309
R^2		0.145	0.0363	0.0449	0.0346
δ		1.19	0.532	0.603	0.458

Tables 16, 17, 18, 19, 20, 21 and 22 show the effect of interaction on, respectively, colleagues, friends, families, three male, two male, two female and three female triads. Interaction always causes triads to walk slower, closer, more aligned (lower y) and with a larger abreast distance. Anyway, the only observable that always presents clear statistical significance and strong effect size is y. For a better understanding, we can compare pdfs for the colleague case (presenting relatively strong effects for all observables), in Figures 5, 6, 7, 8, for, respectively, V, r, x and y. Although it may be counter intuitive that non-interacting triads walk at a closer (abreast) distance, this can be understood by knowing that by walking in less ordered way, triads decrease their width ([1, 2]).

Table 14: Observable dependence on relation for non-socially interacting triads. Lengths in millimetres, times in seconds.

Relation	N_g^k	V	r	x	y
Colleagues	27	1236 ± 34 (σ=177)	713 ± 33 (σ=174)	1002 ± 53 (σ=276)	910 ± 68 (σ=355)
Families	74	1015 ± 19 (σ=164)	691 ± 18 (σ=154)	845 ± 34 (σ=296)	954 ± 40 (σ=343)
Friends	50	1057 ± 23 (σ=165)	603 ± 16 (σ=110)	903 ± 35 (σ=245)	742 ± 47 (σ=336)
$F_{2,148}$		17.2	7.03	3.2	5.77
p		$1.97 \cdot 10^{-7}$	0.00121	0.0435	0.00387
R^2		0.188	0.0867	0.0415	0.0723
δ		1.32	0.811	0.541	0.624

Table 15: Observable dependence on gender for non-socially interacting triads. Lengths in millimetres, times in seconds.

Gender	N_g^k	V	r	x	y
Three females	23	1045 ± 32 (σ=153)	597 ± 21 (σ=99.6)	905 ± 48 (σ=230)	771 ± 65 (σ=313)
Two females	49	1011 ± 20 (σ=142)	672 ± 19 (σ=133)	816 ± 37 (σ=257)	915 ± 53 (σ=372)
Two males	38	1066 ± 31 (σ=193)	685 ± 26 (σ=161)	907 ± 52 (σ=322)	948 ± 49 (σ=305)
Three males	41	1152 ± 33 (σ=208)	680 ± 27 (σ=175)	961 ± 43 (σ=276)	822 ± 59 (σ=381)
$F_{3,147}$		4.81	1.93	2.08	1.72
p		0.00316	0.127	0.106	0.166
R^2		0.0894	0.038	0.0407	0.0339
δ		0.807	0.619	0.545	0.575

Table 16: Observable dependence on interaction for colleague triads. Lengths in millimetres, times in seconds.

Colleagues	N_g^k	V	r	x	y
Interacting	52	1168 ± 19 (σ= 139)	577 ± 16 (σ= 112)	1156 ± 37 (σ= 262)	510 ± 43 (σ= 310)
Non-interacting	27	1236 ± 35 (σ= 177)	713 ± 34 (σ= 174)	1002 ± 54 (σ= 276)	910 ± 70 (σ= 355)
$F_{1,77}$		3.42	17.2	5.77	26.1
p		0.0683	$8.5 \cdot 10^{-5}$	0.0187	$2.32 \cdot 10^{-6}$
R^2		0.0425	0.183	0.0697	0.253
δ		0.445	0.999	0.577	1.23

Table 17: Observable dependence on interaction for friend triads. Lengths in millimetres, times in seconds.

Friends	N_g^k	V	r	x	y
Interacting	162	1032 ± 12 (σ= 153)	552 ± 9.2 (σ= 117)	990 ± 21 (σ= 271)	563 ± 21 (σ= 268)
Non-interacting	50	1057 ± 24 (σ= 165)	603 ± 16 (σ= 110)	903 ± 35 (σ= 245)	742 ± 48 (σ= 336)
$F_{1,210}$		0.973	7.39	4.08	14.9
p		0.325	0.00709	0.0448	0.000153
R^2		0.00461	0.034	0.019	0.0662
δ		0.16	0.442	0.328	0.627

Table 18: Observable dependence on interaction for family triads. Lengths in millimetres, times in seconds.

Families	N_g^k	V	r	x	y
Interacting	322	981 ± 8.5 (σ= 153)	607 ± 8.4 (σ= 150)	886 ± 14 (σ= 252)	707 ± 21 (σ= 380)
Non-interacting	74	1015 ± 19 (σ= 164)	691 ± 18 (σ= 154)	845 ± 35 (σ= 296)	954 ± 40 (σ= 343)
$F_{1,394}$		2.88	18.6	1.48	26.2
p		0.0907	$2.05 \cdot 10^{-5}$	0.225	$4.82 \cdot 10^{-7}$
R^2		0.00725	0.045	0.00374	0.0624
δ		0.219	0.557	0.157	0.661

Table 19: Observable dependence on interaction for three male triads. Lengths in millimetres, times in seconds.

Three males	N_g^k	V	r	x	y
Interacting	90	1141 ± 19 (σ= 180)	575 ± 13 (σ= 118)	1046 ± 30 (σ= 283)	570 ± 34 (σ= 321)
Non-interacting	41	1152 ± 33 (σ= 208)	680 ± 28 (σ= 175)	961 ± 44 (σ= 276)	822 ± 60 (σ= 381)
$F_{1,129}$		0.0938	16	2.54	15.2
p		0.76	0.000108	0.113	0.000158
R^2		0.000726	0.11	0.0193	0.105
δ		0.0582	0.76	0.303	0.74

Table 20: Observable dependence on interaction for two male triads. Lengths in millimetres, times in seconds.

Two males	N_g^k	V	r	x	y
Interacting	135	992 ± 13 (σ= 151)	623 ± 13 (σ= 153)	917 ± 23 (σ= 263)	718 ± 30 (σ= 350)
Non-interacting	38	1066 ± 32 (σ= 193)	685 ± 26 (σ= 161)	907 ± 53 (σ= 322)	948 ± 50 (σ= 305)
$F_{1,171}$		6.18	4.7	0.0382	13.4
p		0.0139	0.0315	0.845	0.000341
R^2		0.0349	0.0268	0.000223	0.0725
δ		0.46	0.401	0.0361	0.675

Table 21: Observable dependence on interaction for two female triads. Lengths in millimetres, times in seconds.

Two Females	N_g^k	V	r	x	y
Interacting	182	962 ± 10 (σ= 134)	593 ± 11 (σ= 143)	887 ± 19 (σ= 254)	682 ± 28 (σ= 374)
Non-interacting	49	1011 ± 20 (σ= 142)	672 ± 19 (σ= 133)	816 ± 37 (σ= 257)	915 ± 54 (σ= 372)
$F_{1,229}$		4.99	12	2.98	14.9
p		0.0265	0.000627	0.0859	0.000148
R^2		0.0213	0.0499	0.0128	0.061
δ		0.361	0.56	0.279	0.624

Table 22: Observable dependence on interaction for three female triads. Lengths in millimetres, times in seconds.

Three Females	N_g^k	V	r	x	y
Interacting	129	1024 ± 13 (σ= 145)	550 ± 11 (σ= 122)	979 ± 24 (σ= 272)	566 ± 28 (σ= 314)
Non-interacting	23	1045 ± 33 (σ= 153)	597 ± 21 (σ= 99.6)	905 ± 49 (σ= 230)	771 ± 67 (σ= 313)
$F_{1,150}$		0.397	3.01	1.49	8.22
p		0.529	0.0848	0.224	0.00474
R^2		0.00264	0.0197	0.00984	0.0519
δ		0.144	0.395	0.278	0.653

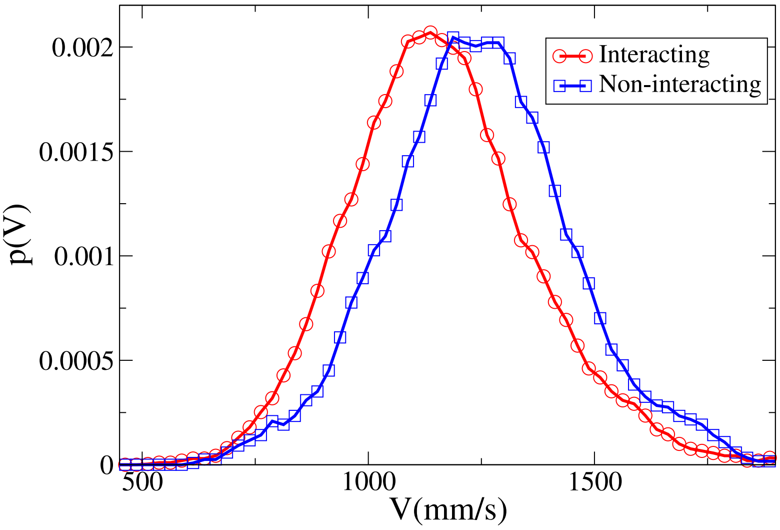

Figure 5: Pdf of the V observable in colleague triads: interacting (red, circles) and non-interacting (blue, squares).

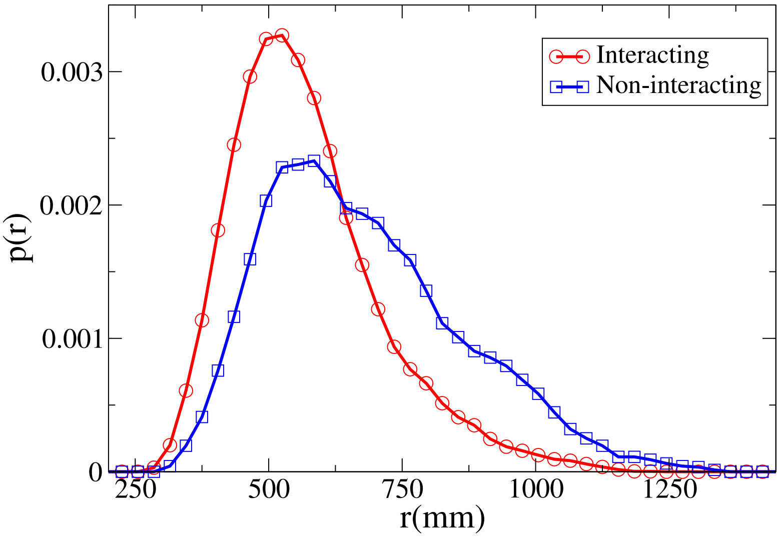

Figure 6: Pdf of the r observable in colleague triads: interacting (red, circles) and non-interacting (blue, squares).

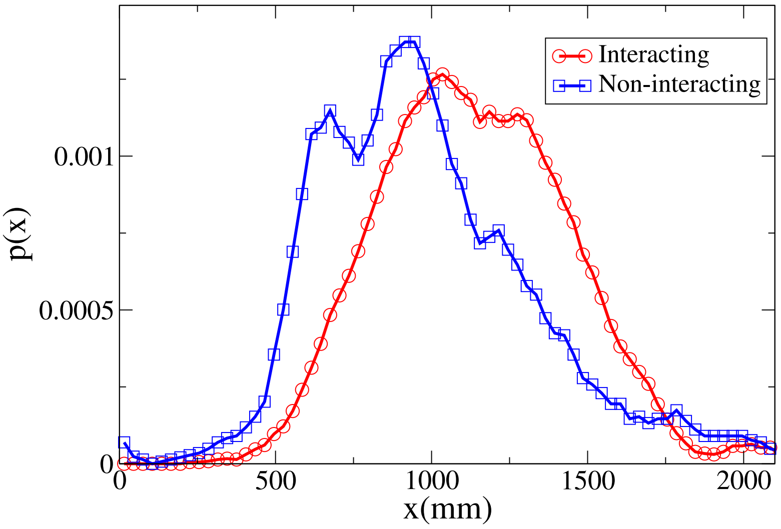

Figure 7: Pdf of the x observable in colleague triads: interacting (red, circles) and non-interacting (blue, squares).

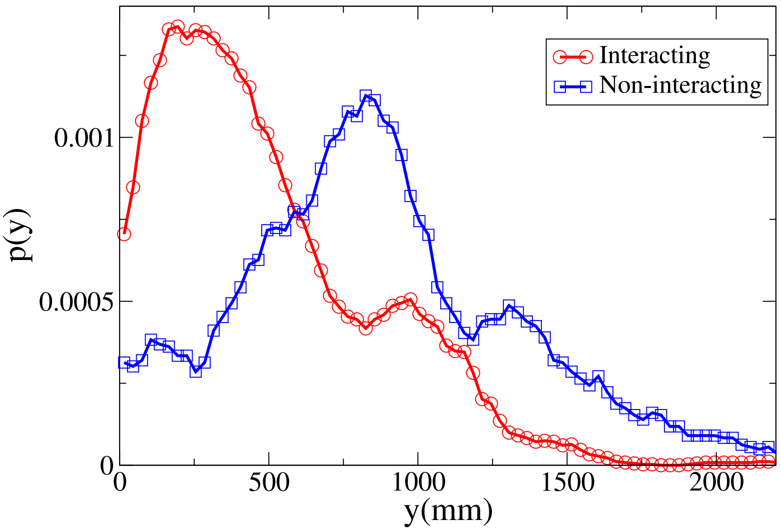

Figure 8: Pdf of the y observable in colleague triads: interacting (red, circles) and non-interacting (blue, squares).

Relation of abreast extension (width) and density

It is interesting to verify whether the tendency of non-interacting triads to have a more limited abreast extension x is affected by density. Tables 23, 24 and 25 show the observable dependence on interaction in, respectively, the fixed $0 \leq \rho < 0.05$ ped/m^2, $0.1 \leq \rho < 0.15$ ped/m^2 and $0.2 \leq \rho < 0.25$ ped/m^2 ranges. Furthermore, Tables 26 and 27 show the p and δ (effect size) values for interaction at different density ranges. Comparing also to Table 12 in the main text, we may see that x results to be smaller for non-interacting triads regardless of density, but the effect appears to be enhanced at higher density. Also the patterns for V, r and y are present at all densities, but effects sizes decrease with density for velocity, while they increase with density for spatial observables.

Table 23: Observable dependence on interaction for triads in the $0 \leq \rho < 0.05$ ped/m^2 range. Lengths in millimetres, times in seconds.

Interaction	N_g^k	V	r	x	y
Interacting	104	1090 ± 16 (σ=163)	628 ± 15 (σ=152)	1121 ± 29 (σ=296)	643 ± 37 (σ=376)
Non-interacting	18	1159 ± 39 (σ=165)	688 ± 43 (σ=181)	1104 ± 78 (σ=332)	806 ± 82 (σ=347)
$F_{1,120}$		2.65	2.17	0.047	2.89
p		0.106	0.144	0.829	0.0915
R^2		0.0216	0.0177	0.000391	0.0236
δ		0.419	0.379	0.0558	0.438

Table 24: Observable dependence on interaction for triads in the $0.1 \leq \rho < 0.15$ ped/m^2 range. Lengths in millimetres, times in seconds.

Interaction	N_g^k	V	r	x	y
Interacting	217	1011 ± 9.9 (σ=145)	577 ± 8.5 (σ=125)	894 ± 17 (σ=246)	649 ± 23 (σ=333)
Non-interacting	85	1059 ± 18 (σ=166)	654 ± 15 (σ=142)	883 ± 31 (σ=282)	848 ± 35 (σ=321)
$F_{1,300}$		6.13	21	0.104	22.1
p		0.0139	$6.84 \cdot 10^{-6}$	0.747	$3.99 \cdot 10^{-6}$
R^2		0.02	0.0653	0.000346	0.0685
δ		0.318	0.588	0.0414	0.603

We report for integrity also the results for dyads in Tables 28, 29, 30, 31 and 32. The patterns are again mostly independent of density (though some exceptions may be found, such as Table 30 presenting a higher y value in interacting dyads), although effect sizes appear to be weaker at high densities. This suggests that in the dyadic case at high densities environmental constraints cause interacting and non-interacting dyads to be more similar. This result may be due to the contrasting tendencies of groups to get a larger x if non-interacting, and at the same time to get a smaller x as density grows.

Table 25: Observable dependence on interaction for triads in the $0.2 \leq \rho < 0.25$ ped/m^2 range. Lengths in millimetres, times in seconds.

Interaction	N_g^k	V	r	x	y
Interacting	83	910 ± 13 (σ=121)	573 ± 16 (σ=147)	810 ± 24 (σ=218)	700 ± 39 (σ=352)
Non-interacting	8	935 ± 34 (σ=97.2)	675 ± 21 (σ=60.7)	653 ± 100 (σ=287)	927 ± 82 (σ=231)
$F_{1,89}$		0.32	3.69	3.47	3.1
p		0.573	0.0578	0.0658	0.0815
R^2		0.00358	0.0398	0.0375	0.0337
δ		0.211	0.716	0.699	0.658

Table 26: p-values for density corresponding to velocity and distance observables for triads at different density ranges. Lengths in millimetres, times in seconds.

Density	V	r	x	y
0-0.05 ped/m^2	0.106	0.144	0.829	0.0915
0.05-0.1 ped/m^2	0.00415	0.00513	0.322	0.000324
0.1-0.15 ped/m^2	0.0139	$6.84 \cdot 10^{-6}$	0.747	$3.99 \cdot 10^{-6}$
0.15-0.2 ped/m^2	0.0171	$6.05 \cdot 10^{-5}$	0.229	$2.15 \cdot 10^{-5}$
0.2-0.25 ped/m^2	0.573	0.0578	0.0658	0.0815

Table 27: Effect size δ-values for density corresponding to velocity and distance observables for triads at different density ranges. Lengths in millimetres, times in seconds.

Density	V	r	x	y
0-0.05 ped/m^2	0.419	0.379	0.0558	0.438
0.05-0.1 ped/m^2	0.423	0.413	0.145	0.533
0.1-0.15 ped/m^2	0.318	0.588	0.0414	0.603
0.15-0.2 ped/m^2	0.379	0.645	0.19	0.685
0.2-0.25 ped/m^2	0.211	0.716	0.699	0.658

Table 28: Observable dependence on interaction for dyads in the $0 \leq \rho < 0.05$ ped/m^2 range. Lengths in millimetres, times in seconds.

Interaction	N_g^k	V	r	x	y
Interacting	415	1146 ± 10 (σ=209)	823 ± 12 (σ=254)	682 ± 7.5 (σ=152)	337 ± 16 (σ=316)
Non-interacting	99	1247 ± 21 (σ=211)	1000 ± 34 (σ=334)	722 ± 26 (σ=255)	528 ± 42 (σ=422)
$F_{1,512}$		18.5	34	4.05	25.2
p		$2.01 \cdot 10^{-5}$	$< 10^{-8}$	0.0446	$7.03 \cdot 10^{-7}$
R^2		0.0349	0.0622	0.00785	0.047
δ		0.482	0.653	0.226	0.563

Table 29: Observable dependence on interaction for dyads in the $0.1 \leq \rho < 0.15$ ped/m^2 range. Lengths in millimetres, times in seconds.

Interaction	N_g^k	V	r	x	y
Interacting	300	1145 ± 10 (σ=179)	749 ± 10 (σ=177)	618 ± 8.1 (σ=141)	309 ± 14 (σ=241)
Non-interacting	82	1252 ± 19 (σ=176)	858 ± 24 (σ=217)	660 ± 21 (σ=186)	395 ± 36 (σ=325)
$F_{1,380}$		23.3	22.1	5.02	6.86
p		$1.97 \cdot 10^{-6}$	$3.59 \cdot 10^{-6}$	0.0256	0.00916
R^2		0.0579	0.055	0.013	0.0177
δ		0.604	0.588	0.28	0.327

Table 30: Observable dependence on interaction for dyads in the $0.15 \leq \rho < 0.2$ ped/m^2 range. Lengths in millimetres, times in seconds.

Interaction	N_g^k	V	r	x	y
Interacting	73	1073 ± 19 (σ=165)	738 ± 21 (σ=180)	561 ± 15 (σ=127)	361 ± 32 (σ=275)
Non-interacting	24	1142 ± 37 (σ=183)	747 ± 28 (σ=135)	602 ± 25 (σ=125)	311 ± 47 (σ=232)
$F_{1,95}$		2.93	0.0448	1.82	0.636
p		0.0899	0.833	0.18	0.427
R^2		0.03	0.000472	0.0188	0.00665
δ		0.408	0.0503	0.321	0.189

Table 31: p-values for density corresponding to velocity and distance observables for dyads at different density ranges. Lengths in millimetres, times in seconds.

Density	V	r	x	y
0-0.05 ped/m^2	$2.01 \cdot 10^{-5}$	$< 10^{-8}$	0.0446	$7.03 \cdot 10^{-7}$
0.05-0.1 ped/m^2	$3.21 \cdot 10^{-7}$	$< 10^{-8}$	0.015	$3.31 \cdot 10^{-8}$
0.1-0.15 ped/m^2	$1.97 \cdot 10^{-6}$	$3.59 \cdot 10^{-6}$	0.0256	0.00916
0.15-0.2 ped/m^2	0.0899	0.833	0.18	0.427
0.2-0.25 ped/m^2	0.91	0.888	0.851	0.863

Table 32: Effect size δ-values for density corresponding to velocity and distance observables for dyads at different density ranges. Lengths in millimetres, times in seconds.

Density	V	r	x	y
0-0.05 ped/m^2	0.482	0.653	0.226	0.563
0.05-0.1 ped/m^2	0.481	0.667	0.227	0.52
0.1-0.15 ped/m^2	0.604	0.588	0.28	0.327
0.15-0.2 ped/m^2	0.408	0.0503	0.321	0.189
0.2-0.25 ped/m^2	0.101	0.13	0.167	0.158

References

[1] Zanlungo F, Brščić D and Kanda T, *Spatial-size scaling of pedestrian groups under growing density conditions* Physical Review E 91 (6), 062810 (2015)

[2] Zanlungo F, and Kanda T, *A mesoscopic model for the effect of density on pedestrian group dynamics*, EPL (Europhysics Letters), 2015; 111, 38007.
