# Supplementary material for: Intrinsic group behaviour II: On the dependence of triad spatial dynamics on social and personal features; and on the effect of social interaction on small group dynamics
Source: PLoS One. 2019 Dec 3;14(12):e0225704. doi: 10.1371/journal.pone.0225704 (PMC6890182; doi:10.1371/journal.pone.0225704)
Supplement: Attachment — Submitted filename: rebuttal.pdf [file pone.0225704.s011.pdf]

We thank our editor and reviewers for their thorough evaluation. We have addressed all the points raised in the reviews. In what follows, we deal with each comment from the Journal (Jou) the editor (Edt) and reviewers (Rev-1, Rev-2), separately. The original comments are given in blue, and our responses are given in black.

On behalf of all authors,
Francesco Zanlungo

Jou-1. We noted in your submission details that a portion of your manuscript may have been presented or published elsewhere:

As stated in the cover letter, this work is a follow-up to our 2017 PloS One publication.

Zanlungo F, et al.,
"Intrinsic group behaviour: Dependence of pedestrian dyad dynamics on principal social and personal features",
PLoS One, 12, 11, e0187253 (Public Library of Science, 2017)

For the sake of integrity, we copied some tables concerning the behaviour of dyads from the aforementioned publication, to which we compare the new results concerning the behaviour of triads. When we present material from the 2017 publication, we always clearly state this fact in the text.

Please clarify whether this [conference proceeding or publication] was peer-reviewed and formally published. If this work was previously peer-reviewed and published, in the cover letter please provide the reason that this work does not constitute dual publication and should be included in the current manuscript.

The work was obviously peer reviewed as it has been published on PLOS. It appears to us that citing explicitly relevant results from previous works, also from the same authors, does not constitute dual publication and it is a normally accepted scientific procedure.

Jou-2. Please provide additional details regarding obtaining participant consent from the coders who participated in your study. In the ethics statement in the Methods and online submission information, please ensure that you have specified (1) whether consent was informed and (2) what type you obtained (for instance, written or verbal, and if verbal, how it was documented and witnessed).

We added new supplementary material including information on coder training and guidance, as required by Rev-1.6, and included in it also information concerning coders' consent. The new supplementary material is introduced at lines 136 and 210 as Annex S1. We also modified the order of the appendices since we noticed that they did not appear in numerical order in the text.

Edt-1. Both Reviewers believe that this manuscript is of potential interest for PLoS one, after minor revisions. Although I do not necessarily agree with every single comment of Reviewer #1, I believe that s/he raises important interpretation issues, and the authors should expand the discussion of their results, following the Reviewer #1's suggestions. The suggestions of Reviewer #2 should be straightforward to implement.

We have expanded the discussion of our results according to the suggestions of Rev-1, and implemented Rev-2 suggestions.

Rev-1.1 Reviewer #1: Title:
In its current form it is too broad and not informative enough. It should be more specific like the title of the previous work on dyads. (i.e.: group behavior in the physical space / intrinsic properties of moving pedestrian triads, etc.) It should express that the present study is about how individual and relational features in triads influence their behavior in the physical space.

We modified the title of our paper as follows "Intrinsic Group Behaviour II: on the Dependence of Triad Spatial Dynamics on Social and Personal Features; and on the Effect of Social Interaction on Small Group Dynamics"

Rev-1.2 The manuscript is poorly placed in context, especially when it comes to the scientific contribution. The study claims that groups presence influences the dynamics of the crowd, moreover, it is assumed that the behavior of these groups may have an impact on evacuation time and the planning of buildings. However, it is not clear or even mentioned on the level of assumptions, how the findings of this research contribute to understand how the presence and dynamics of the groups under observation influence the dynamics of the crowd; and how it could contribute to evacuation plans in emergency situations. As a consequence, the purpose and the scientific contribution of the study is unclear.

From the comment of the reviewer we understood that our explanation of these issues was not sufficient and we tried to expand it.

Current research shows that crowds are mainly composed of groups (cited work [6] even suggests that 85% of pedestrians move in groups, although the figure may be due to the nature of the observed event, a religious gathering in Germany). It then appears clear that any quantitative model of crowds should include group models. Although a few computational models of group behaviour have been introduced, the ecological data [14] on which such models should be based is still mostly missing. As most current pedestrian models are microscopic, in order to be able to reflect diversity in individual behaviour, we believe that providing ecological data (and corresponding statistical analysis) on group diversity we are contributing to the development of such models. Concerning the relevance of group behaviour on egress, we refer to works such as [8-9]. It looks reasonable that the nature of relation in the group should be relevant in attachment during egress, although obviously quantitative ecological data is missing. Although egress behaviour cannot be extrapolated from normal behaviour, we believe that providing information on normal behaviour may be of help. Regarding the quantitative impact of the presence of groups in high-density settings, we refer to our computational work [10]. Since this work, presented at the 2019 Traffic and Granular Flow Conference, has still not been published, we uploaded a preprint on arXiv.

To clarify points more clear we modified our manuscript at lines 32-40 and 47-55.

Rev-1.3 Line 116: The behaviour of walking groups depends also on its intrinsic properties. Age, gender and height are known to affect walking speed (as observed in studies with subjects [38]). The listed properties are not possessed by the group but by the individual group members. At other points of the manuscript, these features are also referred to as group properties incorrectly. Age composition, for example, would be a better choice.

Thank you for pointing out our poor terminology. We corrected the manuscript at lines 69-70 and 86-88.

Rev-1.4 Line 69: ..data set and described how spatial structure, size and velocity of dyads (two people groups) depend on intrinsic properties of groups, and more specifically on:...
The size of dyads is incomprehensible in this way.
Also, the term size / group size is used in an ambiguous way several times (i.e.: line 95) in the introduction, since its exact meaning in the given context is defined much later in the text. Using the term before conceptualization is ambiguous and misleading.

Thank you again for pointing out our poor terminology. We corrected the manuscript at lines 64, 79, 276, 285, 294 and 297.

Rev-1.5 Line 81: Nevertheless, they provided a useful and quantitative insight into how intrinsic features affect dyadic behaviour. The manuscript does not say anything about how and why this insight is useful.

See our answer to comment Rev-1.2.

Rev-1.6 Did human coders receive any training, written guidance including explicit rules for coding? If so, it is supposed to be attached to the annexes.

We added new supplementary material (Annex S1, introduced on lines 136 and 210) with all the relevant information. Referring to the Annex, we also introduced some new information on coding criteria at lines 134-139, 198 and 209-217.

Rev-1.7 `Result section:`
`Line 301 Based on the analysis, triads are always slower than dyads for any values of all intrinsic or extrinsic properties. However, this result is hardly explainable with these properties. A (side)note about the potential effect of group size and coordination strategies should be added or noted as an important aspect that is not detailed in the present study.`
`It would be useful to reflect on the results briefly, not only show the table and merely describe what we can see there, but add a thought / assumption or just play with the idea of what could explain these findings (i.e.: Why can we see high values for group depth in families?)`

We introduced our interpretation of these results at lines 326-335, and 386-389. Consider also our reply at your comment Rev-1.12.

Rev-1.8 `Discussion:`
`The term intrinsic properties is put into quotation marks in the abstract and discussion section while it is present without quotation marks in other parts of the manuscript. Also, in the discussion part, (line 471) ordered groups is used for the first time, and it is unclear what accounts for an ordered group in this context.`
`The discussion is also lacking of presenting how the findings on triads behavior in physical space contribute to a deeper understanding of crowd dynamics and the evacuation of a building. The absence of explanation makes this connection vague and forced.`

Concerning "intrinsic properties", we corrected the manuscript at lines 1, 113 and 496. Concerning "ordered" groups, we explained the term at line 345. Finally, concerning the relevance of the findings to crowd dynamics, refer to our answer to your comment Rev-1.2.

Rev-1.9 `Typographical and grammatical errors`
`Line364  capital D for differences in the beginning of the sentence.`
`Table 8: Observable dependence on minimum age for dyads is in the Height section  should be in the Age section.`

"differences" was actually in the middle of a sentence, but it appeared to be at the beginning of a paragraph because the sentence was split in two by a table. We could modify our latex file to force tables to be in specific positions, but this would probably create problems in the final production of the manuscript by the journal (considering also that the current version is without figures, and thus forcing the position of tables at the current stage does not look appropriate). Similarly, Table 8 is located in the correct section in our manuscript, but appears to be in the Height section because we did not try to force its position. We are confident that these issues will be managed at the production level.

Rev-1.10 `In the Effect of interaction section, the Table 11. and 12. should be swapped for the sake of consistency, as tables on triads are followed by the ones on dyads at every other part of the manuscript.`

Up to Table 10, results concerning dyads were simply citations of results obtained in [46]. Starting from Table 11, results concerning dyads have never been published. When presenting unpublished material, we prefer to follow the dyad-triad convention as in our previous works.

Rev-1.11 `References`
`Reference number 10 (He L, Pan J, Wang W, Manocha D. Proxemic group behaviors using reciprocal multi-agent navigation. In: Proceedings of the International Conference on`

Robotics and Automation. IEEE; 2016. p. 292297.) is not used/flagged in the manuscript. (Most of the used references correspond to the reference list of the authors previous work on dyads.)

Thank you for pointing out this mistake. The work is now cited at line 57 as [15].

Rev-1.12 In general: The manuscript is technically sound. A great amount of ecological data coupled with the achieved high inter-coder reliability is a convincing start for meaningful results. The authors performed an appropriate and rigorous statistical analysis. The data supports the conclusion, and the manuscript is aligned with PLOS Data Policy. It is written in standard English.

At the same time, it is clear that first data analysis took place and then, the framework was constructed around the data, that it is not fortunate to present it such explicitly. The authors properly describe the tables and figures displaying the results, but there is no attempt for interpretation not even to share a few assumptions/ideas about how we should understand and place these findings to a comprehensive picture about group behavior in physical space. This is missing both from the introduction and the discussion part, as well as, a clear and proper connection of the present findings to the claimed purpose of the study.
The introduction is poorly constructed since several terms are used ambiguously. While the reader gets to the point where these terms such as size or relation between members are properly conceptualized and operationalized, they probably get tired and confused by the inconsistency. The authors make these terms clear later, but the other way around would be much more appreciated.
They correctly draw attention both to the potential cultural limitations and the weakness of the apparent belonging approach. Since most of the findings are not surprising but supporting common sense, it would be beneficial to enrich the study by framing them in a colorful way, so it is embedded into a comprehensive image about group behavior in the physical space.

Thank you for the positive opinion on the manuscript. Concerning the aspects of our paper that you criticise, we tried to correct them as pointed out in the replies to the previous comments. Concerning the relative lack of interpretation of results, we would like to add a further comment. As certified also by our previous works, we are very active in using the data that we collect to develop and test theoretical models, and also in using the data and the models in practical applications such as recognition algorithms and human-robot interaction. Nevertheless, we believe that when introducing a new and large data-set as we do in this work, it is better to limit ourselves to present the data and their statistical analysis. We believe that if we act in such a way, the data may be used in an unbiased fashion by other researchers.

Rev-2.1 The authors investigate triads walking dynamics. They study how aspects including age, gender, heights of the group members, together with their relation with each other and the purpose of their trip has an effect on group velocity and distance between group members. The analysis is based on a large-scale data set collecting trajectories of pedestrians within a large multi-purpose building in Osaka. The article is a follow-up of a previous work on the walking dynamics of dyads that two of the authors published in Physical Review E.

Understanding pedestrian walking dynamics is important to plan buildings and emergency evacuation, and the topic may be of interest for the broad readership of PLOS One. The article is clearly written and the statistical analyses have been performed rigorously. I judge the article is suitable for publication in PLOS One, pending the minor changes below.

Thank you for your positive opinion on our work.

Rev-2.2 As a general comment, while the authors investigate in depth the role played by each individual property of the group, they do not dig into the interplay between different factors. For example, one could test a multilinear regression analysis, and study the relative importance of the different variables (for example via Lindeman, Merenda and Gold)

The reviewer raises a very interesting and relevant point. We tried to include an analysis of the interplay between factors in Appendix S3. We did it by considering fixed categories (e.g., colleagues) and analysing how their dynamics depended on given factors (e.g., gender). This analysis is confined to the interplay between two factors, due to the limited extent of the data set and to the natural unbalance in composition of ecological data. Regarding the suggested method (multilinear analysis), we are not confident that a linear analysis would be very suited to the problem, as coefficients of determination are not very good even in the 1D case. We will nevertheless try to cope with this problem in our future works.

Rev-2.3 `Below, there are some minor comments:`
`Typo, line 37: For this reasons -> For these reasons`

We corrected at line 41.

Rev-2.4 `Sentence lines 133-138: Please cut this sentence into multiple sentences.`

We modified at lines 149-154.

Rev-2.5 `Line 132: Please add some more information about how participants were recruited and what do you mean by multi-purpose building.`

We explained the nature of the ATC building at lines 154-156. The data base is entirely composed of ecological data, i.e., the pedestrians were uninstructed users of the ATC centre. Details on consent and ethics may be found at lines 165-170.

Rev-2.6 `Line 212: Add references to Cohens kappa and Krippendorfs alpha`

Thank you for noticing the lack of citations in the main text (they were present only in the related appendix). We added them at lines 233-234.

Rev-2.7 `Line 278: Please define O.`

We provided a better definition at lines 298, 300-301.

Rev-2.8 `Line 281: Please give a quick definition of the presented metrics (ANOVA p-value, effect size, coefficient of determination).`

We explicitly wrote that all metrics are defined in Appendix S4 (lines 305-306).

Rev-2.9 `Table 1 (and all tables): provide more explications in the table captions`

We improved our explanation of table contents at lines 298-307, and included a full explanation also in the caption of the first table (Table 1).
